# An HLA-I signature favouring KIR-educated Natural Killer cells mediates immune control of HIV in children and contrasts with the HLA-B-restricted CD8+ T-cell-mediated immune control in adults

**DOI:** 10.1371/journal.ppat.1010090

**Published:** 2021-11-18

**Authors:** Vinicius A. Vieira, Emily Adland, David F. G. Malone, Maureen P. Martin, Andreas Groll, M. Azim Ansari, Maria C. Garcia-Guerrero, Mari C. Puertas, Maximilian Muenchhoff, Claudia Fortuny Guash, Christian Brander, Javier Martinez-Picado, Alasdair Bamford, Gareth Tudor-Williams, Thumbi Ndung’u, Bruce D. Walker, Veron Ramsuran, John Frater, Pieter Jooste, Dimitra Peppa, Mary Carrington, Philip J. R. Goulder

**Affiliations:** 1 Department of Paediatrics, University of Oxford, Oxford, United Kingdom; 2 HIV Pathogenesis Programme, Doris Duke Medical Research Institute, Nelson R Mandela School of Medicine, University of KwaZulu-Natal, Durban, South Africa; 3 Basic Science Program, Frederick National Laboratory for Cancer Research, National Cancer Institute, Frederick, MD and Laboratory of Integrative Cancer Immunology, Center for Cancer Research, National Cancer Institute, Bethesda, Maryland, United States of America; 4 Department of Statistics, TU Dortmund University, Dortmund, Germany; 5 Nuffield Department of Medicine, University of Oxford, Oxford, United Kingdom; 6 IrsiCaixa AIDS Research Institute, Badalona, Spain; 7 CIBER en Enfermedades Infecciosas, Madrid, Spain; 8 Max von Pettenkofer Institute & Gene Center, Virology, National Reference Center for Retroviruses, LMU München, Munich, Germany; 9 German Center for Infection Research (DZIF), partner site Munich, Munich, Germany; 10 Infectious Diseases and Systemic Inflammatory Response in Pediatrics, Infectious Diseases Unit, Department of Pediatrics, Sant Joan de Déu Hospital Research Foundation, Barcelona, Spain; 11 Center for Biomedical Network Research on Epidemiology and Public Health (CIBERESP), Madrid, Spain; 12 Department of Pediatrics, University of Barcelona, Barcelona, Spain; 13 Translational Research Network in Pediatric Infectious Diseases (RITIP), Madrid, Spain; 14 University of Vic-Central University of Catalonia (UVic-UCC), Vic, Spain; 15 Catalan Institution for Research and Advanced Studies (ICREA), Barcelona, Spain; 16 Germans Trias i Pujol Research Institute (IGTP), Badalona, Spain; 17 Great Ormond Street Hospital for Children NHS Foundation Trust, London, United Kingdom; 18 UCL Great Ormond Street Institute of Child Health, London, United Kingdom; 19 Department of Infectious Diseases, Imperial College, London, United Kingdom; 20 Africa Health Research Institute (AHRI), Durban, South Africa; 21 Ragon Institute of MGH, MIT and Harvard, Cambridge, Massachusetts, United States of America; 22 Max Planck Institute for Infection Biology, Chariteplatz, Berlin, Germany; 23 Division of Infection and Immunity, University College London, London, United Kingdom; 24 School of Laboratory Medicine and Medical Sciences, College of Health Sciences, University of KwaZulu-Natal, Durban, South Africa; 25 Centre for the AIDS Programme of Research in South Africa (CAPRISA), University of KwaZulu-Natal, Durban, South Africa; 26 Oxford NIHR Biomedical Research Centre, Oxford, United Kingdom; 27 Department of Paediatrics, Kimberley Hospital, Kimberley, South Africa; University of Wisconsin, UNITED STATES

## Abstract

Natural Killer (NK) cells contribute to HIV control in adults, but HLA-B-mediated T-cell activity has a more substantial impact on disease outcome. However, the HLA-B molecules influencing immune control in adults have less impact on paediatric infection. To investigate the contribution NK cells make to immune control, we studied >300 children living with HIV followed over two decades in South Africa. In children, HLA-B alleles associated with adult protection or disease-susceptibility did not have significant effects, whereas Bw4 (p = 0.003) and low *HLA-A* expression (p = 0.002) alleles were strongly associated with immunological and viral control. In a comparator adult cohort, Bw4 and *HLA-A* expression contributions to HIV disease outcome were dwarfed by those of protective and disease-susceptible HLA-B molecules. We next investigated the immunophenotype and effector functions of NK cells in a subset of these children using flow cytometry. Slow progression and better plasma viraemic control were also associated with high frequencies of less terminally differentiated NKG2A+NKp46+CD56^dim^ NK cells strongly responsive to cytokine stimulation and linked with the immunogenetic signature identified. Future studies are indicated to determine whether this signature associated with immune control in early life directly facilitates functional cure in children.

## Author summary

In adults, immune control of HIV is strongly influenced by antiviral CD8+ T-cell responses restricted by ‘protective’ HLA class I molecules, such as HLA-B*57, and by ‘disease-susceptible’ HLA class I molecules such as HLA-B*58:02. By contrast, Natural Killer (NK) cells responses make a smaller, albeit significant, contribution. In this study, we evaluate in children living with HIV the contribution of NK cell responses to immune control of HIV, in an age group where HIV-specific CD8+ T-cell responses have less impact on disease outcome. A cohort of >300 therapy-naïve children living with HIV shows that a genetic signature favouring a KIR-education on NK cells is associated with slow progression and better viraemic control. Consistent with this, we observed control of HIV viraemia and lower total HIV DNA levels among children was associated with a less differentiated NKG2A+NKp46+ CD56^dim^ NK cell population that functionally was highly responsive to cytokine stimulation. Thus, the study identifies a signature that can impact future therapeutic strategies to achieve remission in children.

## Introduction

It has been proposed that functional cure of HIV, where HIV is not eradicated but viral RNA in plasma is undetectable after antiretroviral treatment (ART) interruption, may be easier to achieve in children compared with adults [1]. The rationale for this is three-fold. First, in the case of infants infected *in utero*, it is feasible to initiate ART immediately after birth in all children born to mothers living with HIV, and since most intrauterine infections arise late in pregnancy [2], ART can be started very early in the course of infection in these children. In consequence, the size of the viral reservoir is very low and of narrow diversity [3,4]. The second factor is the tolerogenic early life immune environment, with high levels of immune regulation and low activation [5–7]. This reduces the capacity of HIV, even after infection has occurred, to establish a large viral reservoir. A third factor is the replicative capacity of virus transmitted from mother-to-child, which overall is lower in viruses transmitted to the infant than in viruses circulating in the mother [8,9]. The replicative capacity of transmitted virus has been shown in adult transmission studies to have a profound impact on immune activation, proviral HIV-DNA load, and outcome in the recipient [10].

Several case reports in children living with HIV who received ART early in infection and subsequently achieved functional cure have been reported [11–13], but it is clear that early initiation of ART alone is rarely successful in achieving functional cure without other interventions [14,15]. In non-human primates, broadly neutralising antibodies (bnAbs) have facilitated the attainment of functional cure following SHIV infection either when given early following infection in the absence of any ART [16] or when used in combination with a TLR7 agonist during ART [17]. “Shock-and-kill” strategies proposed to achieve cure in HIV infection have identified HIV-specific T-cells as playing a critical role in clearance of HIV-infected cells after latency reversal [18–20] and it has been reported that bnAbs can augment the antiviral T-cell efficacy through a vaccinal effect [16]. However, it remains unclear which effector cells are likely to facilitate functional cure in paediatric infection, since Th1-supporting cytokines do not approach adult levels until the second decade of life [21–23].

In adult HIV infection, the major immunogenetic factors contributing to immune control in African populations are the ‘protective’ HLA-B molecules such as HLA-B*57/58:01/81:01, and the ‘disease-susceptible’ HLA-B molecules such as HLA-B*18:01/45:01/58:02 [24–27]. HLA-C expression levels also affect disease outcome via CD8+ T-cell activity [28]. *HLA-A* expression influences immune control via biasing education of Natural Killer (NK) cells, as low *HLA-A* expression alleles are associated with slower progression and better viraemic control in adults [29] by reducing surface expression of HLA-E, the ligand for the inhibitory CD94/NKG2A receptor that forms the non-polymorphic arm of NK cell education. *HLA-B* expression levels also not vary by allele and its surface density influences NK cell responsiveness via KIR3DL1 interaction [30]. HLA-E expression on the cell surface is also dependent of the presence of Threonine (T) or Methionine (M) at position -21 in the signal peptide (the -21M variant binds to HLA-E) of HLA-B alleles [31]. HLA class I (HLA-I) molecules expressing the Bw4 motif in combination with its Killer cell immunoglobulin-like receptor (KIR) ligand (KIR3DL1) licence NK cells to kill in the absence of HLA-I [30] and are associated with slower HIV disease progression in adults [32–39].

The complexity of these interactions is revealed by the segregation of HLA-I haplotypes in two distinct groups with a direct consequence on the NK cell education program [40]. Eleven of 12 HLA-B Bw4 alleles are also -21T and therefore do not provide the peptide for HLA-E. All HLA-C molecules bind to a KIR and the dimorphism in the residues 77 and 80 of the α1 helix differentiates the HLA-C molecules into C1 and C2 groups, promoting their binding with KIR2DL2/L3 and KIR2DL1, respectively [41]. C2 alleles provide stronger KIR binding than C1 alleles and more than 92% of the C2 haplotypes are in linkage disequilibrium with -21T HLA-B alleles. The haplotypes encoding -21M HLA-B are Bw6 and C1, providing good expression of HLA-E but not KIR binding, favouring NK cell inhibition via NKG2A. Due to linkage disequilibrium, -21M HLA-B haplotypes have not only a low diversity of C1 alleles, but they are enriched with those with a low level of expression. This group is highly dominated by HLA-C*07, which is affected by microRNA-148a downregulation, leading to poor KIR binding [42]. On the other hand, haplotypes encoding -21T HLA-B can offer both HLA-C1 and -C2 alleles, and also the Bw4 motif, increasing the amount of KIR ligands and decreasing the supply of peptides to HLA-E [40]. This clear segregation creates two distinct education programs that affect the diversity of NK cells and their ability to sense abnormalities, as well their effector function and response to diseases.

In paediatric HIV infection, the ‘protective’ and ‘disease-susceptible’ HLA-B molecules that strongly affect adult immune control have a much more modest effect on outcome [8]. Although HIV-specific CD8+ T-cell responses are detectable from birth [43,44], they generally impose weak selection pressure on the virus and lack sufficient breadth through the childhood years to have a substantial impact on viraemia [45]. Less is known about the part played by NK cells in immune control of HIV in early life and the potential for NK based immune interventions to facilitate HIV remission in paediatric infection. In the VISCONTI cohort of adult post-treatment controllers, the HIV-specific CD8+ T-cell responses were no more potent than those of non-controllers, and the cohort was enriched for disease-susceptible HLA-I, suggesting a mechanism independent of strong cytotoxic T-cell lymphocyte (CTL) activity [46]. Subsequent studies in this cohort have suggested that an immunogenetic signature reflecting KIR-educated NK cells and a corresponding NK cell phenotype and functionality may mediate functional cure in these subjects [47]. Studies in African green monkeys, natural hosts of SIV, support the possible role of NK cells in purging lymphoid tissue sanctuary sites of virus-infected cells that are inaccessible to CD8+ T-cells [48,49]. Recent studies of early-ART-treated human infants infected with HIV demonstrate correlations between NK cell responses and intact proviral reservoirs at only 12 weeks of age [4]. Also, as observed in adults, KIR3DL1-HLA-Bw4 has been associated with lower plasma viral load and higher CD4+ T-cell counts in all ages in paediatric infection [50]. Together, these findings suggest the possibility that early life NK responses may have better potential than HIV-specific T-cell activity to control viral replication, and therefore to contribute also to functional cure in early-ART-treated children.

To investigate this question, we studied a historical cohort of >300 children living with HIV from sub-Saharan Africa that we have followed for up to two decades, to determine the major immunogenetic factors influencing disease outcome in children compared with an adult cohort in KwaZulu-Natal, South Africa. We then investigated the functional aspects of the NK cell responses in a representative subset of these children.

## Results

### HLA-Bw4 and *HLA-A* mRNA expression levels are associated with slow-progression in children

**Fig 1:**
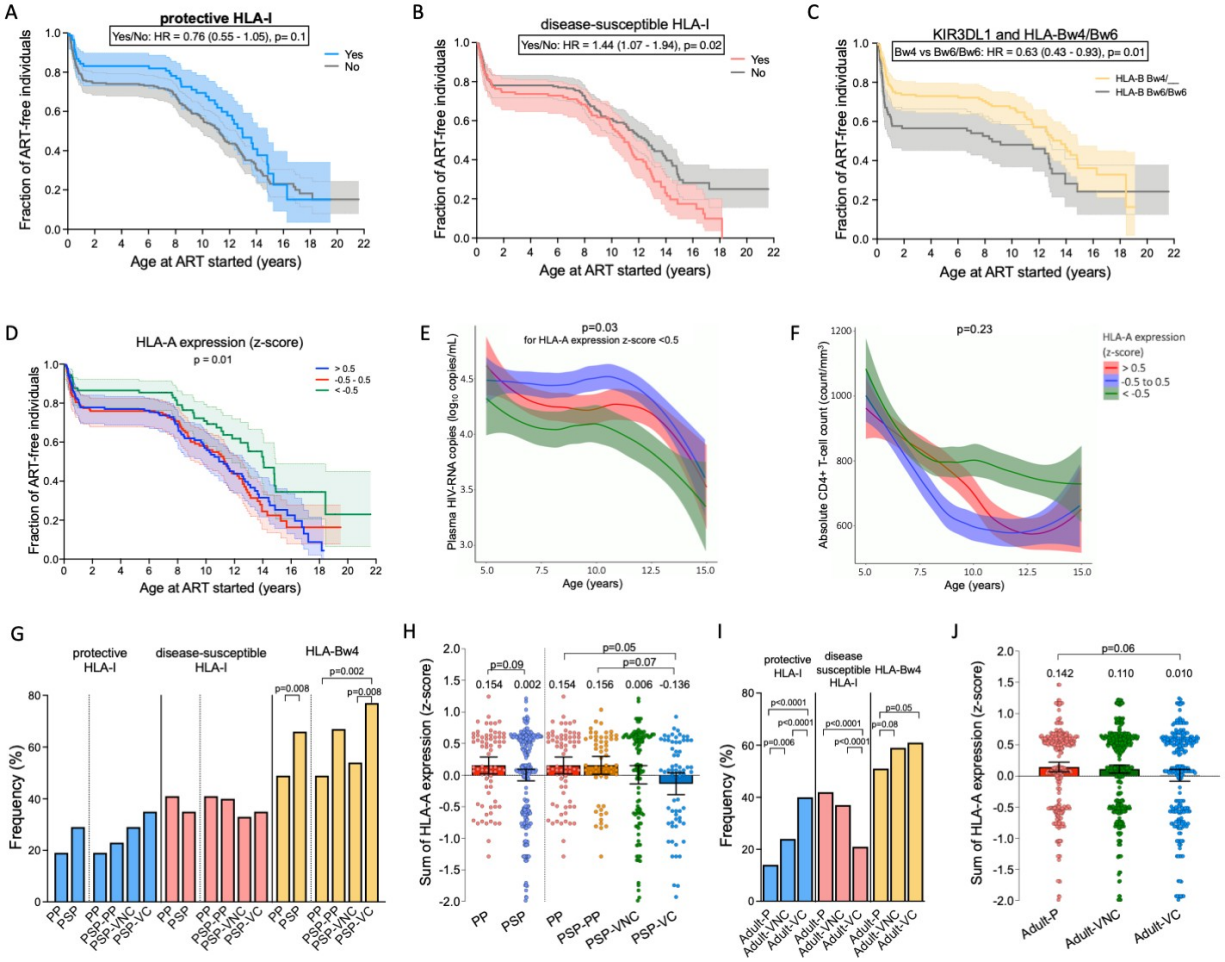
HLA class I and KIR interaction in paediatric and adult HIV infection. A to D. Effect of protective HLA-B*57/58:01/81:01 (A), disease-susceptible HLA-B*18:01/45:01/58:02 (B), HLA-Bw4 (C), and *HLA-A* expression z-score (D) on time to start ART due to progression (absolute and relative CD4+ T-cell count <350 cells/mm^3^ and/or < 20%). Data was censored if ART initiated without immunological progression. Hazard Ratio (HR) and p-values are based on log-rank comparisons between the presence or not of the listed HLA-I alleles. E and F. Impact of *HLA-A* expression z-score on longitudinal plasma HIV-RNA load (E) and absolute CD4+ T-cell count (F) in children older than five years old. The best fit line and the 95% confidence interval are shown in red for z-score > 0.5, blue from -0.5 to 0.5, and green for <-0.5. p-value was obtained from the linear mixed-effects modelling comparing the three groups. G. Frequency of the disease-protective and the disease-susceptible HLA-I and HLA-Bw4 alleles in each group. Comparisons were based on Chi-square test and corrected with Bonferroni-Holm due to multiple-comparison tests. H. HLA-A numeric z-score of each individual is plotted. Statistical comparison between two groups were based on unpaired t-test and between the four groups on ANOVA followed by Tukey’s test for multiple comparisons. I and J show similar analyses for the adult cohort. PP = paediatric progressor; PSP = paediatric slow-progressors; PSP-VC = PSP viraemic controllers; PSP-VNC = PSP viraemic non-controller; Adult-P = adult progressor; Adult-VNC = adult viraemic non-controllers, Adult-VC = adult viraemic controller.

To evaluate the impact of HLA and KIR on disease outcome in paediatric HIV infection, we studied 310 children living with HIV from sub-Saharan Africa, 97% of whom were followed in clinics in South Africa and the remaining 3% followed in clinics in London and Barcelona. Using, as the primary outcome, time to start ART as a result of meeting the prevailing immunological WHO criteria for ART initiation at the time of follow-up (absolute and relative CD4+ T-cell count < 350 cells/mm^3^ and/or < 20%), the presence of protective HLA alleles in HIV-infected children trended towards slower progression without reaching significance (HR = 0.76, p = 0.1; Fig 1A and S1A Fig), while disease-susceptible HLA alleles were significantly associated with faster progression (HR = 1.44, p = 0.02; Fig 1B). The effect of HLA-B*18:01 and HLA-B*45:01 was most marked in the first 5–6 years of life (S1B Fig). Thus, on the basis of these univariate analyses, both protective and disease-susceptible HLA appear to have some impact (albeit not statistically significant in the case of protective HLA-B alleles) on HIV disease progression in paediatric infection that is consistent with the HLA effects observed in adult infection.

The combination of HLA-Bw4 and KIR3DL1 has been associated with slow progression in HIV infection in both adult [32–39] and paediatric [50] studies. In our paediatric cohort also, individuals with at least one HLA-Bw4 allele progressed significantly slower than HLA-Bw6/Bw6 individuals (HR = 0.63, p = 0.01, Fig 1C). Since almost all individuals in the cohort express KIR3DL1 (S1 Table), the presence or absence of KIR3DL1 did not affect the analyses. Of note, the adult defined disease-susceptible HLA-B*58:02 is a Bw4 allele and its detrimental effect in accelerating progression is only observed after the age of 10 years in children (S1B Fig). We did not type the KIR3DL1 alleles and could not evaluate the synergistic effect of HLA-Bw4 with high expressing KIR3DL1 alleles observed in adults [35].

The levels of HLA-A expression are stable across individuals [29]. Previously, the levels of *HLA-A* mRNA levels were measured using quantitative PCR in large cohorts of Caucasian and African individuals and further validated in a cohort of South African subjects living with HIV—the detailed methodology and the estimated *HLA-A* expression values have been published [29] (S2 Table). We summed, for each study subject, the expression levels of the two *HLA-A* alleles using the previously published data to evaluate the role of *HLA-A* expression on disease progression in the paediatric cohort. We observed an association between low-expressing *HLA-A* alleles and slow progression to meet criteria to initiate ART (Fig 1D, p = 0.01). Mixed-effects regression analyses of longitudinally followed children aged > 5 years who had not met the prevailing criteria to initiate ART showed a lower plasma viral load in children with the lowest *HLA-A* z-scores (p = 0.03, Fig 1E) when compared to the group with highest z-scores and similar trend but less clear-cut benefits of low *HLA-A* expression on absolute CD4+ T-cell count (Fig 1F). Due to the low frequency of -21M homozygous HLA-B alleles in this cohort, we were not able to see any effect of -21M/M in our analyses, as previously has been demonstrated in adults [29].

We next categorised the paediatric cohort into paediatric progressors (PP, n = 73) and paediatric slow progressors (PSP, n = 237), according to whether they had met immunological criteria to initiate ART according to the guidelines at the time of follow-up (relative and absolute CD4+ T-cell count < 20% and/or <350 cells/mm^3^) before 5 years of age, and subdivided the PSP group in three further subgroups: PSP who eventually progressed to meet CD4 criteria to initiate ART between 5 and 10 years of age (PSP-PP, n = 57); and paediatric slow-progressors who did not meet criteria to start ART by 10 years, who were either relative viraemic non-controllers (PSP-VNC: plasma viral load >10,000 HIV RNA copies/ml, n = 112) or relative viraemic controllers (PSP-VC: viral load <10,000 HIV RNA copies/ml, n = 68) (S1 Table and S2 Fig). Neither the protective nor the disease-susceptible HLA-B alleles were significantly enriched in any of these groupings (Fig 1G), apart from HLA-B*57 when comparing PP with PSP and PSP-VC (p = 0.05 and p = 0.03, respectively; S3A and S3B Fig). HLA-Bw4 was more frequent in the PSP and PSP-VC groups (p = 0.008 and p = 0.002 compared to PP, Fig 1G), and HLA-A expression was also lower among PSP-VC than PP and PSP-PP (p = 0.05 and p = 0.07, Fig 1H).

To compare these immunogenetic effects of HLA and KIR in the paediatric cohort with those observed in adults, we conducted similar analyses in a group of 998 ART-naïve, chronically infected HLA-I typed adults from South Africa, of whom 405 were also KIR typed. Adults living with HIV in the chronic phase of infection were included if they had at least three plasma viral loads and CD4+ T-cell counts spanning a year. The median follow-up time was 3.6 (IQR 1.3–5.6) years. As adult viraemic non-progressors with normal absolute CD4+ T-cell counts are very rarely seen [51,52], we classified the adult population according to their immunological progression and viral control in adult progressors (Adult-P: absolute CD4 <200 cells/mm^3^, n = 288), adult viraemic non-controllers (Adult-VNC, absolute CD4>200 cells/mm^3^, plasma viral load >10,000 RNA copies/mL, n = 449) and adult viraemic controllers (Adult-VC, absolute CD4 >200 cells/mm^3^, viral load <10,000 RNA copies/ml, n = 260), as similarly done in other previous studies [33,35]. Although the protective and the disease-susceptible HLA-I are very strongly associated with immunological and virological status (Fig 1I), the impact of HLA-Bw4 alleles and HLA-A expression levels appeared substantially more modest (Fig 1I and 1J); the opposite to what was observed in the paediatric cohort.

### Regression analyses indicate stronger influence of HLA-A and Bw4 than HLA-B on outcome in children

**Fig 2:**
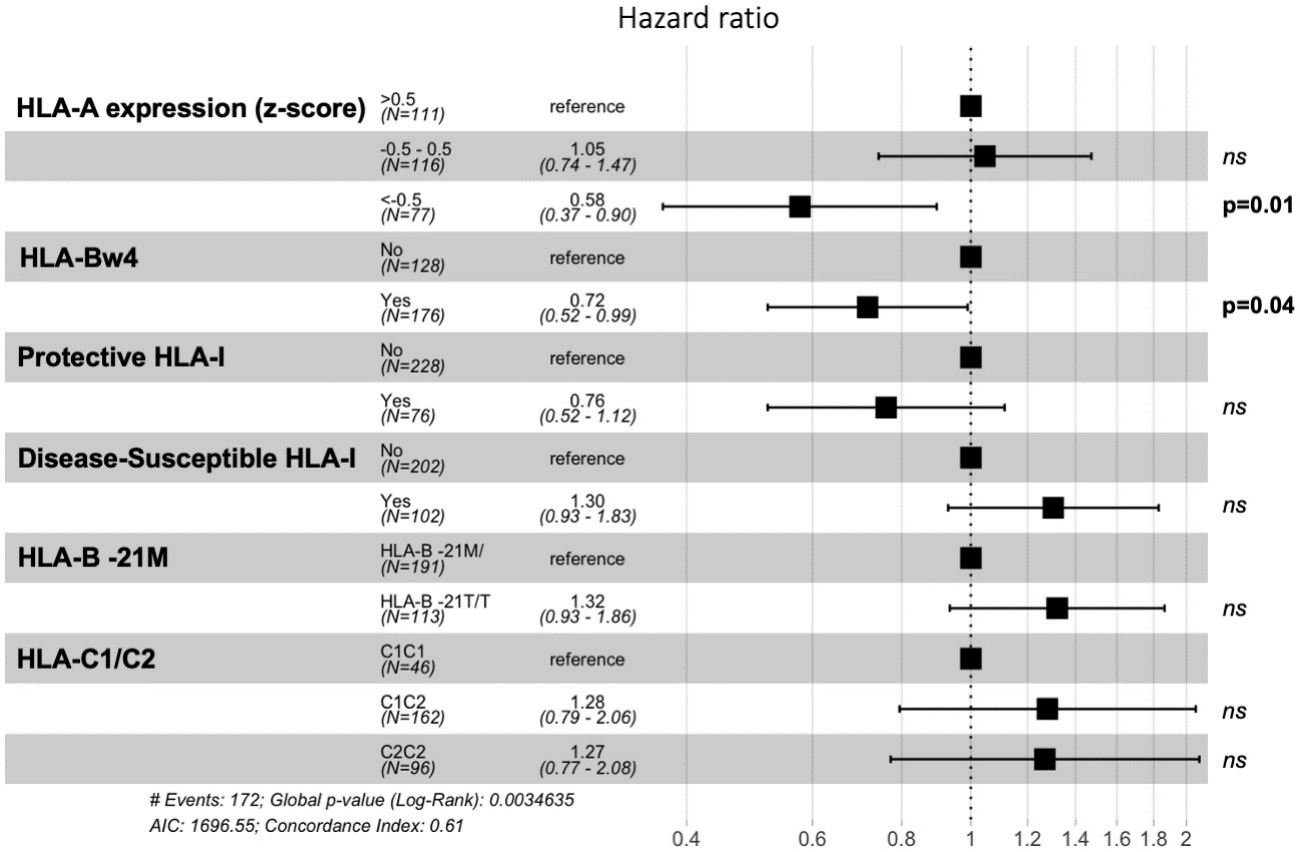
Evaluation of immunogenetic factors on time to start ART. Cox-proportional hazard model evaluating the effect of protective and disease susceptible HLA-I alleles, HLA-Bw4, HLA-B -21T/M, HLA-C1/C2 and *HLA-A* expression level on time to start ART due to progression (absolute and relative CD4+ T-cell count <350 cells/mm^3^ and/or < 20%). Data was censored if ART initiated without immunological progression.

To estimate the relative impact of each of the immunogenetic factors studied on HIV disease outcome in children and adults, we first used a Cox proportional-hazard model to determine hazard ratios of these parameters for protection against progression to meet CD4 criteria for ART initiation in children (Fig 2). Whilst there was no significant independent effect of the protective or the disease-susceptible HLA-B alleles on outcome, the presence of HLA-Bw4 (p = 0.04) and low *HLA-A* allele expression (p = 0.01) were independently associated with a slower disease progression. We did not observe any clear effect of -21M or T HLA-B alleles or HLA-C1 and C2 alleles. Again, due to the low frequency of -21M homozygous in our cohort, we were not able to acertain its impact on the HIV disease outcome.

We next ran three logistic models including all the covariates, having as outcomes for the paediatric groups in the first model the categorical variables PP and PSP, and, in the second, PP, PSP-PP, PSP-VC and PSP-VNC. For the adult cohort, a third model used as outcomes: Adult-P, Adult-VNC and Adult-VC (Table 1). In both paediatric models, HLA-Bw4 and *HLA-A* expression levels were significantly associated with outcome. Of note, Bw4 remained significant even after adjusting for protective HLA-I alleles and the HLA-A effect being more marked in the second model that included viraemic control among the outcomes. In neither paediatric model did adult-cohort defined protective and disease-susceptible HLA-B have a significant impact on outcome. By contrast, in the adult model, protective and disease-susceptible HLA-B had the strongest effects on outcome. HLA-Bw4 and HLA-A expression had significant albeit lower impact in adults, and less of an impact, according to the odds ratios generated by the models, than in paediatric infection. Similar findings were obtained when individual protective and disease-susceptible HLA-I alleles were considered (S3 Table).

**Table 1:** Logistic regression models in the paediatric and adult cohorts.

**Model 1**. Outcome: PP = 1; PSP = 0						
**Predictor**	**Coefficient**	**Standard error**	**p-value**	**OR**	**95% CI of OR**	
					**Lower**	**Upper**
*Intercept*	0.19266	0.10975	0.08035	1.21	0.98	1.50
Protective HLA-I	-0.06371	0.06079	0.29557	0.94	0.93	1.06
Disease-susceptible HLA-I	0.08180	0.05669	0.15017	1.08	0.97	1.21
HLA-Bw4	-0.17295	0.05742	**0.00285**	**0.84**	**0.75**	**0.94**
HLA-B -21T	0.08933	0.10645	0.40214	1.10	0.89	1.35
HLA-C C1C1	*Reference*					
C1C2	0.07499	0.07783	0.33619	1.08	0.92	1.25
C2C2	0.06030	0.07783	0.33619	1.06	0.90	1.25
HLA-A (z-score)	0.08134	0.03966	**0.04130**	**1.08**	**1.01**	**1.17**
**Model 2**. Outcome: PP = 3, PSP-PP = 2, PSP-VNC = 1, PSP-VC = 0						
**Predictor**	**Coefficient**	**Standard error**	**p-value**	**OR**	**95% CI of OR**	
					**Lower**	**Upper**
*Intercept*	1.36496	0.27711	<0.0001	3.92	2.27	6.74
Protective HLA-I	-0.27350	0.15349	0.07594	0.76	0.56	1.02
Disease-susceptible HLA-I	0.13246	0.14312	0.35555	1.14	0.86	1.51
HLA-Bw4	-0.42740	0.14498	**0.00349**	**0.65**	**0.49**	**0.86**
HLA-B -21T	0.23293	0.26877	0.38693	1.26	0.74	2.14
HLA-C C1C1	*Reference*					
C1C2	0.15877	0.19652	0.41989	1.17	0.80	1.72
C2C2	0.07129	0.21078	0.73546	1.07	0.71	1.62
HLA-A (z-score)	0.30516	0.10015	**0.00255**	**1.35**	**1.12**	**1.65**
**Model 3**. Outcome: Adult-P = 2, Adult-VNC = 1, Adult-VC = 0						
**Predictor**	**Coefficient**	**Standard error**	**P-value**	**OR**	**95% CI of OR**	
					**Lower**	**Upper**
*Intercept*	1.068053	0.85302	<0.0001	2.91	2.46	3.44
Protective HLA-I	-0.270221	0.055879	**<0.0001**	**0.76**	**0.68**	**0.85**
Disease-susceptible HLA-I	0.249808	0.053009	**<0.0001**	**1.28**	**1.16**	**1.42**
HLA-Bw4	-0.142084	0.054087	**0.00875**	**0.87**	**0.78**	**0.96**
HLA-B -21T	0.004181	0.080804	0.95875	1.00	0.86	1.18
HLA-C C1C1	*Reference*					
C1C2	0.020518	0.063699	0.74744	1.02	0.90	1.16
C2C2	0.023111	0.068771	0.7369	1.02	0.89	1.17
HLA-A (z-score)	0.078734	0.033122	**0.01764**	**1.08**	**1.01**	**1.15**

Therefore, the combination of Bw4 alleles and low expressing *HLA-A* alleles decreases the inhibition via NKG2A and favours the education via KIRs of NK cells, these being the immunogenetic factors most strongly associated with immune control in the paediatric cohort.

### A NK cell signature characterised by high NKG2A expression is strongly associated with favourable paediatric outcome

**Fig 3:**
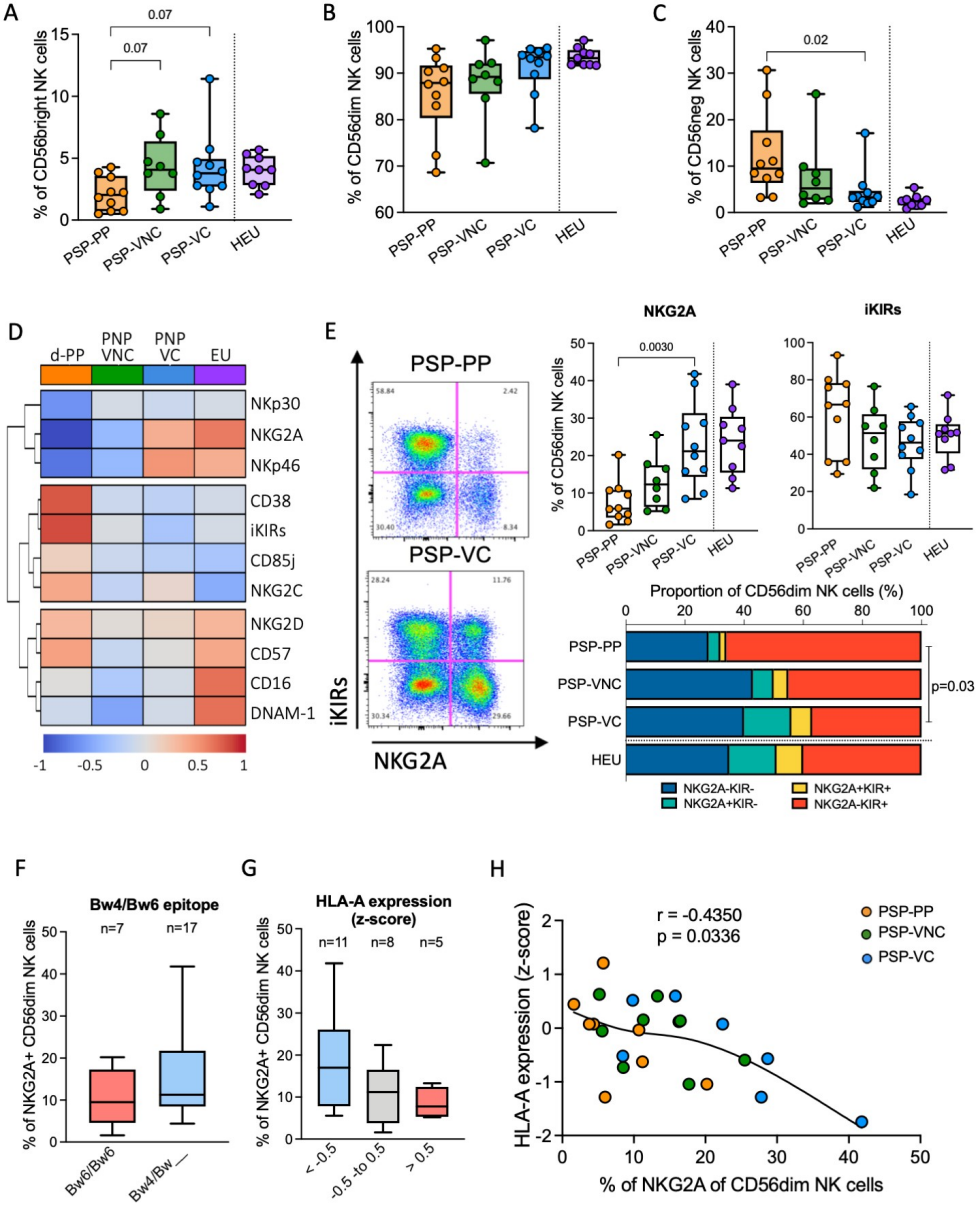
Subset distribution and phenotype of NK cell population. A, B and C. Frequency of CD56^bright^, CD56^dim^ and CD56^neg^CD16+ NK subsets, respectively, among PSP-PP, PSP-VNC, PSP-VC and HEU. Statistical comparison between the three HIV-infected groups was based on Kruskal-Wallis test followed by Dunn’s test for multiple comparisons. D. Heatmap showing the median z-score base on the frequency of surface markers on CD56^dim^ NK cells in each group. E. Typical FACS plot obtained with NKG2A and iKIRs staining associated with the summary for the expression of these markers in each group. Statistical comparison between the three groups was based on Kruskal-Wallis test followed by Dunn’s test for multiple comparisons. Combination of NKG2A and iKIRs fractions in CD56^dim^ NK cell standardized to the total from the median of each subset per group. Statistical comparison based on the permutation test performed by SPICE. F and G. Effect of HLA-Bw4 (F) and *HLA-A* expression z-score (G) on the frequency of NKG2A+ CD56^dim^ NK cells. H. Correlation between NKG2A+ CD56^dim^ NK cells and *HLA-A* expression z-score. Spearman rank test was performed and the black line represents smoothing spline.

The differentiation and functional activity of the NK cell population are influenced by different factors including the immunogenetics and the impact of immunogenetics on the education program, but also age and infections (e.g., HIV and CMV). To investigate the NK cell immunophenotype of the distinct clinical groupings of children living with HIV defined above, we first compared age-matched individuals who fulfilled the criteria for the PSP-PP (n = 10), PSP-VNC (n = 8), and PSP-VC (n = 10) groups (S4 Table). PP were excluded from this analysis due to the very-early stage of progression and ART-initiation. As a reference group, we included HIV-exposed uninfected children/adolescents (HEU, n = 9). In order to take account of the effect of CMV infection on the NK population, only CMV seropositive individuals were included. Although there was no difference in total NK cell frequency (S4 Fig), disease progression was associated with a trend towards a lower frequency of the CD56^bright^ subset and an expanded CD56^neg^ population (Fig 3A–3C, gating strategy in S4 Fig), as previously described in adult studies [53,54]. The PSP-VC had a similar subset distribution to the HEU.

Assessing the surface expression of several markers on the CD56^dim^ subset to better characterize this NK cell population, PSP-VC had a higher frequency of the natural cytotoxicity receptor (NCR) NKp46 on their surface than PSP-PP (p = 0.007) (Fig 3D and S5A–S5H Fig). We did not observe any difference in the other activating markers, such as DNAM-1, NKG2D, NKG2C and CD16. In the heatmap, NCR expression clustered together with NKG2A and these were highly co-expressed in the PSP-VC compared to PSP-PP and PSP-VNC (p = 0.002 and p = 0.06, respectively, S6 Fig). By contrast, CD38, CD85j, NKG2C were clustered together with inhibitory KIRs (iKIRs) expression and tended to be more expressed in the PSP-PP (Fig 3D).

The frequency of NKG2A+ cells was higher in the PSP-VC than in the PSP-PP (p = 0.003), and there was a tendency for iKIRs expression to be lower, although this was not statistically significant (Fig 3E). The NKG2A+ CD56^dim^ NK population, either positive or negative for KIRs, was distinctly expanded in the PSP-VC (p = 0.03) and like HEU. This enrichment of the NKG2A+ population with higher expression of NCRs in PSP-VC demonstrates a population skewed towards a less mature phenotype. Co-expression patterns showed that NKG2A expression alone or in combination with iKIRS, CD85j and CD57 was significantly higher in the PSP-VC, suggesting different stages of NK cell differentiation, whereas no difference was observed in the terminally differentiated iKIRs+CD57+ NK cells (S7 Fig).

Despite the small sample size studied here, we considered whether the immunogenetic signature found in the larger cohort correlated with the NK cell immunophenotype. Indeed, we observed a trend towards a higher frequency of NKG2A+ cells in the participants with HLA-Bw4 and low-expressing *HLA-A* alleles and a higher expression of NKG2A on CD56^dim^ NK cells in association with low *HLA-A* expression (p = 0.03) (Fig 3F–3H). We did not find any effect of -21M/T HLA-B on NKG2A expression, probably as a consequence of the low representation of -21M homozygous individuals (S8 Fig) and the impact of all subjects in our study being CMV seropositive [55]. Together these findings provide further evidence of the influence of inherited genetic factors on the NK cell population’s immunophenotype.

**Fig 4:**
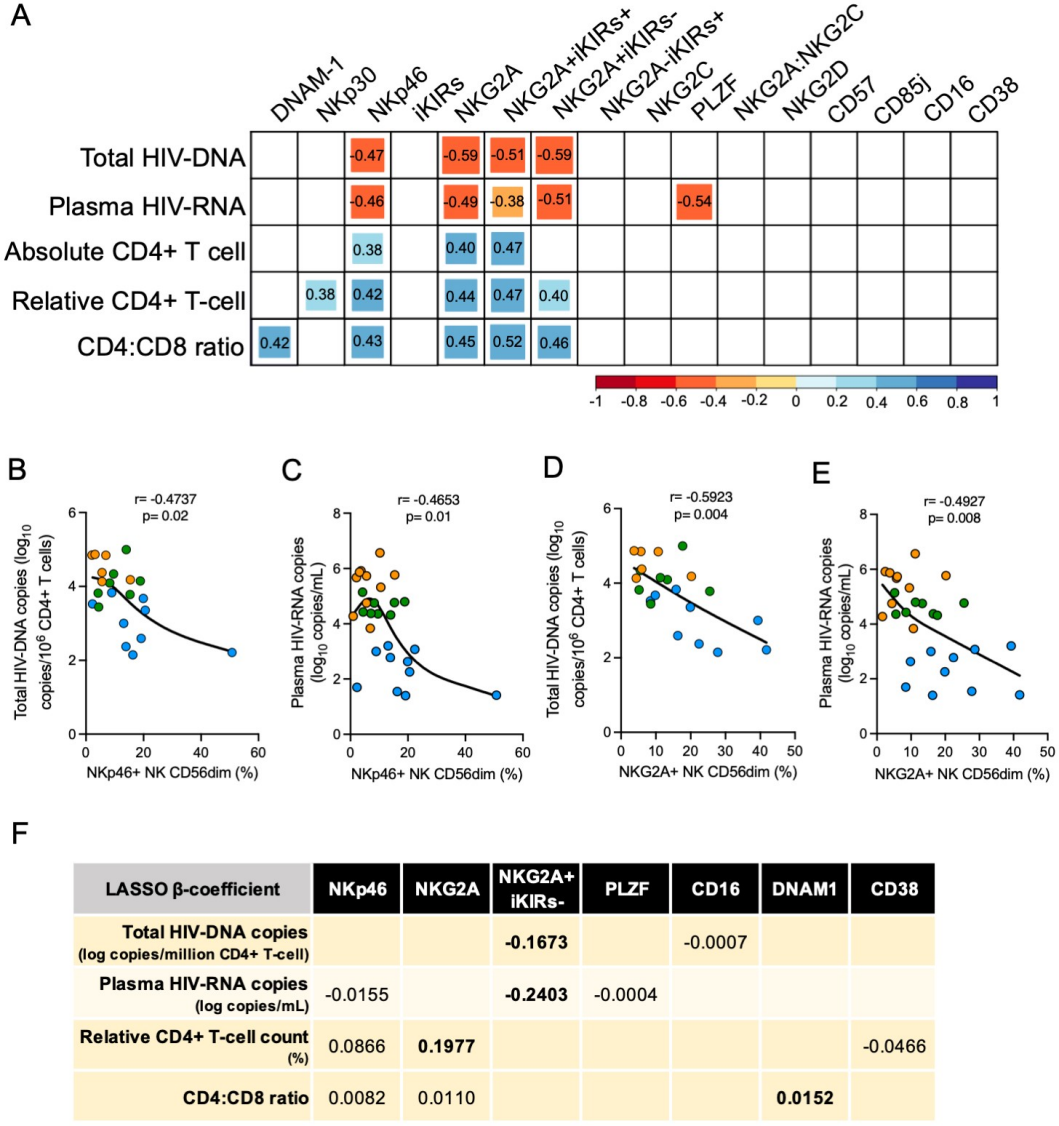
Correlation between different markers on CD56^dim^ NK cells and disease severity. A. Matrix showing only significant correlation between each different marker and total HIV-DNA copies (log_10_ copies/10^6^ CD4+ T-cell), plasma HIV-RNA copies (log_10_ copies/mL), absolute CD4+ T-cell count (count/mm^3^), relative CD4+ T-cell count (%) and CD4:CD8 ratio. Layers of red indicate a negative correlation and layers of blue indicate a positive correlation. Square’s size is proportional to the r value. B to E. Plots showing NKp46+ and NKG2A+ CD56^dim^ NK cell subsets correlations. Spearman rank test was performed for correlations and the black line represents smoothing spline. PSP-PP, PSP-VNC and PSP-VC are shown in orange, green and blue dots, respectively. F. Covariates selected by the LASSO model and their β coefficients. All variables that were significant correlated to the outcome in Fig 6A were included in the final model.

Next, we investigated whether expression of these immunophenotypic parameters on the CD56^dim^ NK subset correlated with markers of immunological HIV disease progression and viraemic control. NKp46 and NKG2A expression both correlated negatively with total HIV-DNA and plasma HIV-RNA copies, and positively with absolute and relative CD4+ T-cell count, and CD4:CD8 ratio (Fig 4A–4E). These associations of NKG2A+ cells were independent of iKIRs. Next, the LASSO principle was used to identify which were the major predicting covariates that contributed to these outcomes (Fig 4F). NKG2A+ or NKG2A+iKIRs- were selected in all models and had the highest impact in the outcome investigated, with the single exception of CD4:CD8 ratio, for which DNAM-1 expression had a similar impact. These findings reinforce the strong relation between the NKG2A+ NK cell population and the favourable immunological and virological outcomes in the paediatric group.

**Fig 5:**
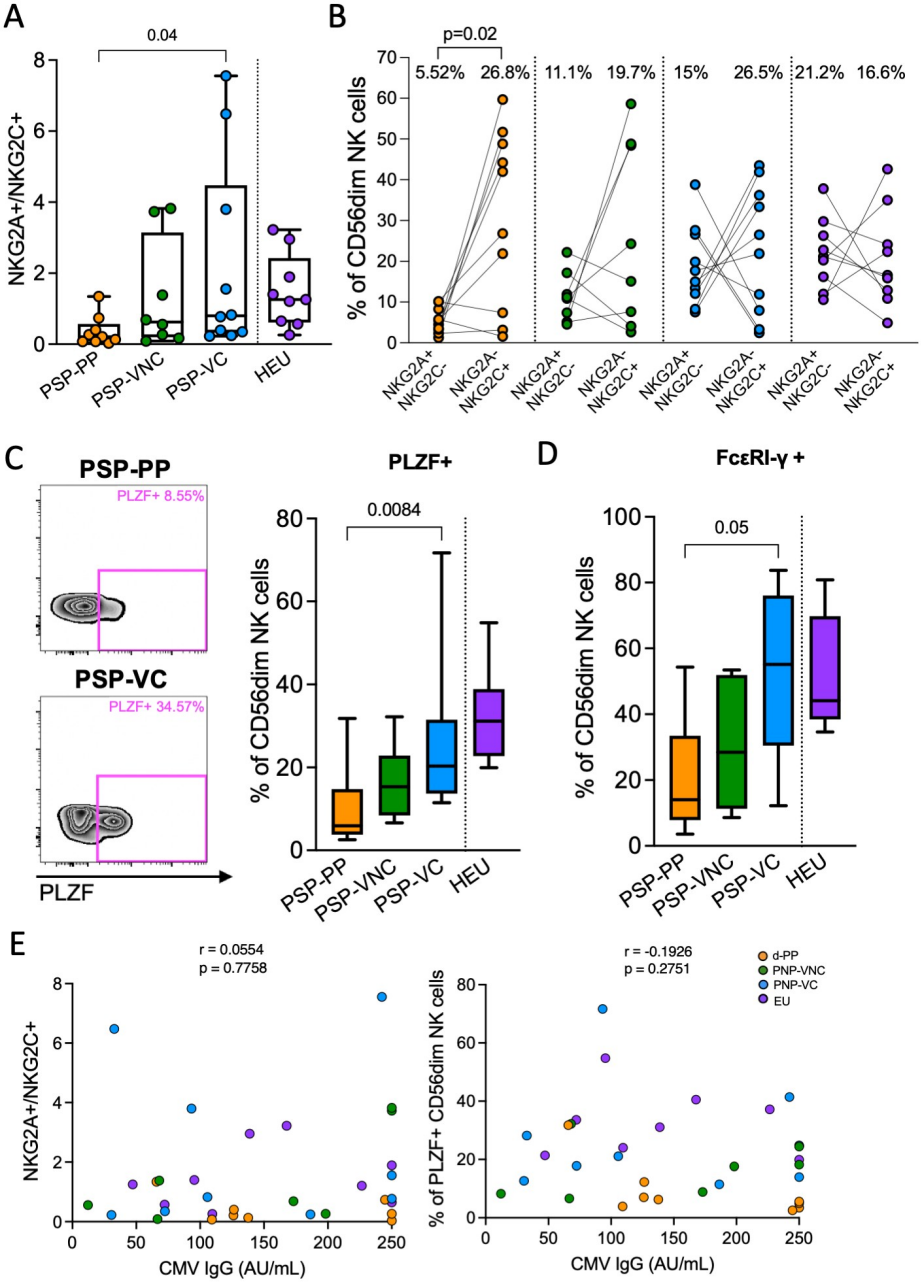
Expansion of memory-like NK cells in PSP-PP. A. NKG2A/NKG2C ratio in each of the four groups. Statistical comparison was based on Kruskal-Wallis test followed by Dunn’s test for multiple comparisons. B. Pairwise comparison of NKG2A+NGK2C- and NKG2A-NKG2C+ subsets in each group. Wilcoxon matched-pairs test was performed to compare the two populations. C and D. Typical FACS plot and frequency of PLZF+ and FcεRI-γ+ in CD56^dim^ NK cell subset in each group. Statistical comparison was based on Kruskal-Wallis test followed by Dunn’s test for multiple comparisons. E. Correlation of CMV IgG levels with NKG2A/NKG2C ratio and PLZF. Spearman rank test are shown for correlations. Simple linear regression best-fit line and 95% CI are shown within the grey area. The coloured dots represent: HEU (purple), PSP-VC (blue), PSP-VNC (green) and PSP-PP (orange).

In contrast to the PSP-VNC and PSP-VC groups, we observed in the PSP-PP a stronger association with lower frequency of the NKG2A+NKp46+ subset, CD56^neg^ expansion and, finally, a higher proportion of memory-like CD56^dim^ NK cells. Although PSP-PP did not show a clear expanded NKG2C+ population (S5H Fig), we found a lower NKG2A:NKG2C ratio and an increase of the NKG2A-NKG2C+ population in this group (Fig 5A and 5B). Moreover, PSP-PP clearly displayed a downregulation of PLZF and FcεRI-γ in the CD56^dim^ population (Fig 5C and 5D), a recognised signature of the memory-like NK cell subset. Although all individuals were CMV positive, we did not observe any correlation between CMV IgG levels and NGK2A:NKG2C ratio or PLZF expression (Fig 5E), as has previously been described in adults [56].

### Enhanced cytokine responsiveness of CD56^dim^ NK cell in viraemic controllers

**Fig 6:**
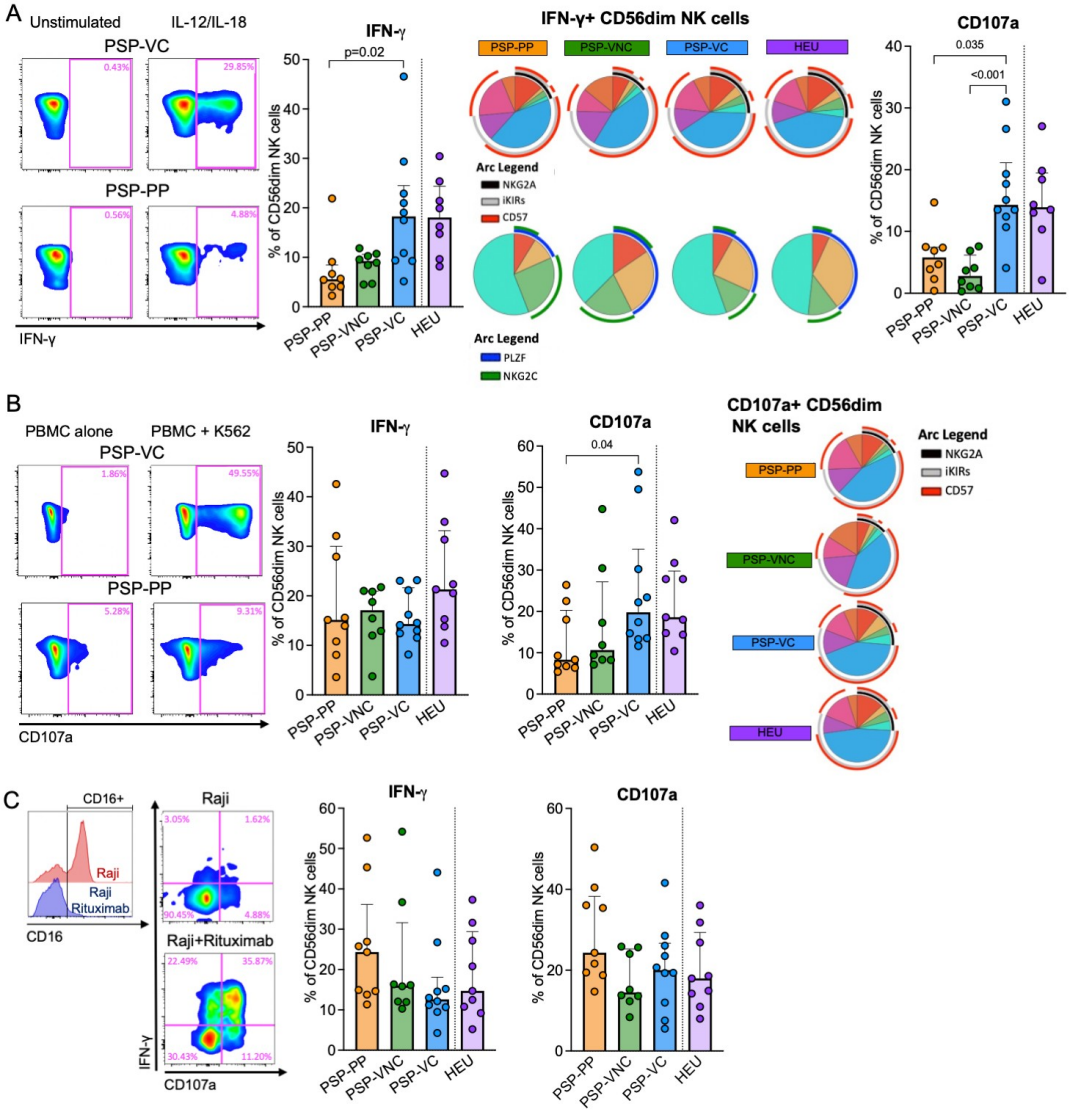
Effector function of CD56^dim^ NK cells. A. Summary of IL-12/IL-18 cytokine stimulation showing typical FACS plots and the frequency of IFN-γ+ of total CD56^dim^ NK cells. Pie charts performed by SPICE looking the combination of NKG2A, iKIRs, and CD57 (top), and NKG2C and PLZF (bottom) on IFN-γ+ CD56^dim^ NK cells. The frequency of cells expressing CD107a is shown on the top right. B. Summary of assays having K562 cell line as target cells showing typical FACS plots and the frequency of CD107a+ and IFN-γ+ of total CD56^dim^ NK cells. Pie charts performed by SPICE looking the combination of NKG2A, iKIRs, and CD57 on CD107a+ CD56^dim^ NK cells. C. Summary of ADCC assay via RAJI as target cells showing engagement of CD16 receptor in the presence of Rituximab (decrease in CD16+ staining, typical FACS plots and frequency of CD107a+ and IFN-γ+ of total CD56^dim^ NK cells. Statistical comparison between the three HIV-infected groups was based on the Kruskal-Wallis test followed by Dunn’s test for multiple comparisons.

To assess the impact this phenotype has on CD56^dim^ NK cell effector function, we evaluated the ability of these cells in the different paediatric subgroups to secrete IFN-γ and degranulate via different pathways. IL-12 and IL-18 stimulation induced strong IFN-γ production in the PSP-VC compared to PSP-PP (p = 0.02, Fig 6A). As expected, a higher proportion of IFN-γ+ cells were NKG2A+ and PLZF+ in these first two groups. The frequency of IFN-γ positive cells correlated positively with the frequency of NKG2A+ CD56^dim^ NK cells, and CD4+ T-cell percentage, while negatively with plasma viral load (S9 Fig). Similarly, we observed higher levels of degranulation (CD107a) in the PSP-VC compared to PSP-PP (p = 0.035) and PSP-VNC (p<0.001).

When incubated with K562, a cell line lacking HLA-I, we did not observe the same difference in the IFN-γ expression. However, a tendency towards higher degranulation was present among the PSP-VC (Fig 6B). Similarly, these two groups tended to have a higher proportion of CD107a+ cells expressing NKG2A. Finally, the ability to perform Antibody-dependent cellular cytotoxicity (ADCC) was assessed in response to stimulation with RAJI cells coated with anti-CD20 (Rituximab). The median percentage of IFN-γ and CD107a was similar between the groups but tended to be higher in the PSP-PP group, which is expected due to the expansion of memory-like phenotype of their NK cell population (Fig 6C).

## Discussion

In this study, we sought to evaluate the major immunogenetic factors influencing HIV disease outcome in children. In an ART-naïve historical cohort of >300 children living with HIV from sub-Saharan Africa, we showed that maintenance of CD4+ T-cell counts and also lower plasma viraemia were significantly associated with low-expressing *HLA-A* alleles and/or with the presence of the HLA-Bw4 motif. However, the HLA-B molecules that in adults confer strong protection against progression, or that are associated with more rapid disease progression, did not significantly contribute to outcome in paediatric infection. This immunogenetic signature associated with better disease outcome in children favours a KIR-education program of the NK cell compartment. The immunophenotypic and functional analyses of NK cells identified a less differentiated population with more plasticity characterised by high NKG2A/NKp46 expression on CD56^dim^ NK cells in viraemic controllers that correlated with low total HIV DNA copies, while NK cells in progressors showed a terminally differentiated phenotype. Finally, the major functional feature distinguishing CD56^dim^ NK cells in slow progressor/low viraemic children is their enhanced ability to produce cytokines and degranulate in response to IL-12/IL-18 stimulation as opposed to ADCC-mediated function.

These findings contrast with the predominant immunogenetic mechanisms of immune control of adult HIV infection. Although *HLA-A* and *HLA-C* expression levels, and HLA-Bw4 in association with KIR3DL1, have important influences on disease outcome, these do not have as great an impact as differences in HLA-B type. The dominant immunogenetic impact of HLA-B on adult HIV outcome is highlighted also by the data shown in the current study. This HLA-B effect is believed to be principally, but not solely, related to the broad, immunodominant Gag-specific CD8+ T cell responses restricted by protective HLA-B molecules such as HLA-B*57, HLA-B*58:01 and HLA-B*81:01 [57–61]. Gag is an important target since it is both highly abundant and conserved in sequence. As a result, Gag escape mutants often inflict a cost on viral replicative capacity [60,62,63]. Other factors contributing to the dominant effect of HLA-B include the ability of HLA-B to bind LILRB2, and thereby influence the innate immune response [64], and the tapasin dependence of HLA molecules, which strongly influences epitope breadth [65].

These data support previous studies showing that differences in HLA-B do not significantly affect plasma viral load in children living with HIV and that even HIV-specific CD8+ T cell responses restricted by protective HLA-B do not successfully control viraemia in early life [8]. As in adults, Gag- and Pol-specific CD8+ T-cell responses in early life contribute somewhat to improved control of viraemia, but a median breadth of only one Gag-specific response over the first 5 years of life in HIV-infected children [45] explains the modest impact on viraemia compared with broad Gag-specific responses generated within weeks of infection observed in adult controllers [60]. Several factors contribute to the narrow response observed in children, notably the low levels of Th1-cell polarising cytokines produced by early-life innate immune cells [66,67] and the vertical transmission of viruses carrying escape mutants pre-selected by the HLA-I molecules shared by the mother [44,68,69]. In adults the speed and extent of CD8+ T-cell activation following infection, and the nature of the HLA-B-restricted HIV-specific CD8+ T-cell response are together strongly associated with viral setpoint [70,71]. As in children, transmission of virus pre-adapted to the HLA class I molecules expressed in the recipient has a very substantial impact on disease outcome in adults [10], but the frequency of this happening is clearly much lower when donor and recipient are unrelated compared to the situation in MTCT when 50% (or more in the case of homozygosity) of HLA class I molecules in the child are shared with the mother.

NK cell responses in infants have been less well-studied than in adults but NK cell numbers and antiviral functions are reportedly similar in neonates and adults and indeed non-cytolytic inhibition of CCR5-tropic HIV replication by neonatal NK cells through the production of MIP-1ß is deemed to be superior [72–75]. Previous studies have demonstrated that HLA-Bw4 in combination with KIR3DL1 is associated with higher CD4+ T-cell counts in children living with HIV, and HLA-Bw4-80I in combination with KIR3DS1 with higher plasma viral loads [50]. Preliminary data from an early-ART-treated cohort of infants in Botswana demonstrated an association between CD56^dim^ NK cells expressing NKp30 and low intact proviral reservoir size within 3 months of birth [4]. Our own data showing a strong presence of Bw4 and of low-expressing *HLA-A* alleles that create an immunogenetic signature favouring NK cell education via KIRs, along with the presence of a NK cell phenotype dominated by expression of NKG2A and NKp46, are associated with better immune and viral control in children. Adding to these findings, the adult disease-susceptible effect of the Bw4 allele HLA-B*58:02 was only observed in children older than 10 years, reinforcing the lower impact of T-cell activity and the protective effect of the Bw4 motif in HIV-disease progression in early life. By contrast the disease-susceptible Bw6 alleles HLA-B*45 and HLA-B*18:01 had a marked detrimental impact during the first 10 years of life.

Although these findings are consistent with an immunogenetic signature favouring a KIR-education program on NK cells in children having a stronger impact on disease outcome than adult-defined HLA-B alleles, nonetheless there is some contribution of the HLA-B alleles that are disease-protective and those that are disease-susceptible to outcome in paediatric infection. In particular, HLA-B*81 is unusual if not unique among protective HLA-B alleles in expressing the Bw6 motif, and HLA-B*81 appears to have some independent protective effect in children (S3 Table), whereas the protective effects of HLA-B*57 are not evident, consistent with the stronger effect of the Bw4 motif expressed. So, while the NK responses appear to have stronger effects in control of viraemia in early life, the HIV-specific CTL responses mediated by protective HLA-B alleles also make some contribution.

A immunogenetic signature favouring a KIR-education program and lower HLA-E surface expression, with consequently decrease in NKG2A engagement, has an impact on the NK cell immunophenotype with a higher frequency of NKG2A+ cells [40]. The fact that the immunogenetics underpin the correlations favouring a KIR-education program and lower HLA-A expression leading to better paediatric HIV disease outcome suggests that the NKG2A-expressing NK cell signature can be at least partially a consequence of the immunogenetics, and has a causal link with immune control of HIV in children, as opposed to being an effect of it. Our findings are consistent with previous work showing that NKG2A-expressing NK cells are superior in cytotoxic and non-cytotoxic activities in HIV-infection [76,77] and in preventing transmission of HIV [78]. The less mature phenotype observed in slow progressors and viraemic controllers favours an NK cell population with higher plasticity and responsiveness to cytokines, while an increase in the more terminally differentiate CD56-CD16+ population was observed in HIV disease progressors. A NK signature expressing NKp46/NKp30 has been associated with better control of viral reservoir [79]. Of note, the process of NK cell differentiation towards a terminally differentiate stage, marked by progressive expression of CD57, occurs parallel and independent to the education process and KIRs acquisition [80]. Therefore, the immunophenotype observed here of NKp46+NKG2A+ in association with better disease outcome in children might not be entirely a consequence of the process of NK cell education driven by the inherited immunogenetic signature. For example, the perturbed homeostasis and altered cytokine environment that may arise in progressors would preferentially drive the terminal differentiation of NK cells and/or immune exhaustion. Thus, whilst the immunophenotypic and functional NK cell pattern associated with immune control of HIV in paediatric infection is consistent with the immunogenetic signature favouring KIR education observed here, factors additional to the genetics can contribute.

We observed a negative correlation between *HLA-A* expression on paediatric HIV outcome with the less mature NKG2A+NKp46+ CD56^dim^ NK cells, but no effect of polymorphism at residue -21 of the signal peptide of HLA-B molecules. -21M variants bind HLA-E and therefore individuals with HLA-B molecules that express the -21M variants could synergise with high-expressing *HLA-A* alleles in facilitating NKG2A education, leading to more NK cell inhibition and less killing of the infected targets [29]. The reasons for this are not well understood but may be related to the differential linkage disequilibrium (LD) patterns that exist between HLA among distinct populations.

Indeed, strong LD exists segregating HLA-I allotypes that support either a KIR- or a NKG2A-education program [40]. The vast majority of HLA-B Bw4 alleles are also -21T and are usually inherited with both HLA-C2 and -C1 alleles, which provides KIR-binding with KIR2DL1 and KIR2DL2/L3, respectively. In contrast, -21M haplotypes are relatively frequent in HLA-B Bw6 alleles, which do not bind KIRs and are highly dominated by the HLA-C*07, an HLA-C1 allele with less strong KIR interaction and affected by microRNA-148a mediated downregulation [42]. Although we easily detected an enrichment of Bw4 alleles, we did not find a higher proportion of HLA-C2 and -21T-HLA-B in slow progressors/viraemic controllers. Interestingly, the HLA-C2 segregation with -21T and Bw4 alleles is also not as clear in African populations and may help explain our findings [40].

The dichotomous effect of -21M/T is less clear in CMV positive populations due to the effect of CMV infection in reshaping the NK population [55]. Our data show an expansion of a memory-like subset in paediatric progressors. Interestingly, albeit all children were CMV positive, we did not observe any near correlation between IgG levels and the frequency of memory-like NK cells, as in adults [56]. Our findings might reflect a role of HIV viraemia/inflammatory environment to imprint the expansion of this phenotype independent of CMV. It seems likely that CMV intermittently reactivates in association with AIDS-progression independently of IgG levels. Also, memory-like NK cells are expanded in HIV-infected adults and correlated with systemic immune activation [81]. This population has specific anti-HIV/SIV activity [82] and was shown to be expanded in secondary lymphoid tissue in non-pathogenic HIV-infection [83]. Although lower in frequency, we cannot exclude their potential activity in paediatric viraemic control in secondary lymphoid tissue.

There are limitations to our study. First, due to faster progression in children and the rarity of viraemic controllers, we do not have a large sample size to detect the possible effect of less frequent genotypes, such as -21M/M. However, we were able to detect a stronger association of KIR-HLA interaction with disease progression and viral control than in adults. Due to the cross-sectional nature of functional aspects of this study, further concerns might be raised about the causality between the less mature NKG2A+ phenotype and slow progression. However, our identification of inherited factors not modifiable by disease course and previously known to influence the distribution and function of the NK cell population supports our findings. We have not demonstrated through functional analyses a specifically effective anti-HIV response in this study. This was not possible owing to unavailability of more samples for these assays. It is important to underline that that these analyses used cryopreserved samples from a historical cohort because now WHO guidelines have recommended that all children living with HIV are treated with ART irrespective of CD4+ T-cell count.

Here, we have shown genetic and phenotype signatures are associated with slower progression and viraemic control in vertically HIV-infected children. The reduced role of the CTLs response in paediatric viraemic control and the full effector potential of NK cells in early life have important implications in functional cure strategies for children living with HIV. In an era of early suppressed children with low levels of viral reservoir, we can identify potential immunogenetic signatures that may increase the chance of remission upon ART interruption where it is expected that the host immune system will play a role in controlling viral reactivation.

## Methods

### Ethics statement

Informed written consent was provided for participation by all individuals or their caregivers if they were underage. This study was approved by the University of KwaZulu-Natal Biomedical Research Ethics Committee and the Oxford Research Ethics Committee.

### Study population

We included 310 vertically and antiretroviral therapy (ART)-naïve children living with HIV of sub-Saharan Africa origin from four different cohorts: Kimberley (South Africa) [8,84], Durban (South Africa) [84,85], London (United Kingdom) [86], and Barcelona (Spain) [86]. The children were categorised as either paediatric progressors (PP) if CD4+ T-cell declined below 20% or absolute CD4 counts was <350 cells/mm^3^ and ART was started before the age of five years, or slow progressors (PSP) if they reached five years old with CD4+ T-cell >350 cells/mm^3^ and > 20% without meeting criteria to initiate ART. The median time to start ART due to immunological progression in the PP group was 0.5 years (IQR 0.3–0.8), while 49% of PSP started ART due to progression at a mean age of 11.2 years (IQR 8.3–13.0).

The PSP group was further subdivided into delayed progressors (PSP-PP) if they met the criteria to initiate ART (CD4+ T-cell <350 cells/mm^3^ or < 20%) before the age of 10 years; in case they did not progress by the age of 10 years, they were classified as viraemic non-controllers (PSP-VNC) or viraemic controllers (PSP-VC) according to whether their median plasma HIV-RNA load during follow-up was above or below 10,000 copies/mL, respectively (S3A–S3C Fig, S1 Table). The CD4+ T-cell value refers to the snapshot of the first CD4 measurement available after five years of age, and the plasma HIV-RNA value is the individual median value after the age of five years when the viral setpoint is achieved in children [84]. PSP-PP had a median plasma HIV-RNA load of 71,095 copies/mL (IQR 28,000–170,623) and a median absolute and relative CD4+ T-cell count of 446 cells/mm^3^ (IQR 383–543) and 16% (IQR 14–18%). PSP-VNC had a median plasma HIV-RNA load of 45,049 copies/mL (IQR 27,000–106,229) and a median absolute and relative CD4+ T-cell count of 619 cells/mm^3^ (IQR 506–910) and 26.5% (IQR 23.3–30.9%). Finally, PSP-VC had a median plasma HIV-RNA load of 2,614 copies/mL (IQR 584–5,762) and a median absolute and relative CD4+ T-cell count of 779 cells/mm^3^ (IQR 568–985) and 30.9% (IQR 26.1–33.3%).

For the flow cytometry assays, representative subjects with PBMC available were selected from the PSP-PP, PSP-VNC and PSP-VC groups. In addition, as a reference and control group, HEU children were selected. Like the subjects living with HIV, HEUs are part of the same cohort followed at Prince Mshiyeni Memorial Hospital (Durban, South Africa). However, the levels of HIV exposure are unknown, and no data were available on plasma viral load and drug suppression of HEU’s mothers.

We also investigated 998 HIV-infected ART-naïve adults from a previously described cohort in South Africa [25,60,87]. Clinical data, plasma HIV-RNA copies, CD4+ and CD8+ T-cell counts were collected as part of their regular follow-up. The adult subjects were subdivided in a similar fashion into three groups: adult progressors (Adult-P), defined as having a sustained absolute CD4+ T-cell count of < 200 cells/mm^3^, viraemic non-controllers (Adult-VNC) (absolute CD4+ T-cell count >200 cells/mm^3^, and plasma HIV-RNA copies >10,000 copies/mL); and viraemic controllers (Adult-VC) (absolute CD4+ T-cell >200 cells/mm^3^, and plasma HIV-RNA <10,000 copies/mL). Adult-P had a median plasma HIV-RNA load of 143,500 copies/mL (IQR 50,300–450,000) and a median absolute CD4+ T-cell count of 161 cells/mm^3^ (IQR 99–192), while Adult-VNC had a median plasma HIV-RNA load of 68,250 copies/mL (IQR 26,675–132,500) and a median absolute CD4+ T-cell count of 360 cells/mm^3^ (IQR 303–496). Adult-VC had a median plasma HIV-RNA load of 2,805 copies/mL (IQR 673–4854) and a median absolute CD4+ T-cell count of 492 cells/mm^3^ (IQR 438–696).

For specific subjects, HCMV IgG levels were measured with ARCHITECT CMVG assay (Abbott Diagnostics, IL, USA) from frozen plasma samples and considered positive if above 15 AU/ml following the recommendations by the manufacturer.

### HLA and KIR genotyping

HLA typing was performed using a targeted next-generation sequencing (NGS) method. Briefly, locus-specific primers are used to amplify HLA-A and B (exons 1 to 4) and C (exons 1 to 5) genes with Fluidigm Access Array (Fluidigm Singapore PTE Ltd, Singapore). The Fluidigm polymerase chain reaction (PCR) amplicons are pooled and subjected to sequencing on an Illumina MiSeq sequencer (Illumina, San Diego, CA 92122 USA). HLA alleles and genotypes are called using the Omixon HLA Explore (version 2.0.0) software (Omixon, Budapest, Hungary).

KIR genotyping for the presence or absence of each KIR gene was conducted by PCR with sequence-specific priming (PCR-SSP) as described previously [88], with some modifications. Each PCR was conducted in a volume of 5 ul using 5 ng genomic DNA and SYBR Green PCR Master Mix with Platinum Taq (Invitrogen). Presence and absence of specific PCR products were detected by melting curve analysis on the 7900 Real-Time PCR System (Applied Biosystems). An algorithm developed in-house assigned KIR genotypes based on melting curve data. Additional 2DS4 subtyping to detect the null variant, which has a 22bp deletion in exon 5, was performed by size discrimination of the 2DS4 PCR products using the LabChip GX instrument (Caliper).

### Total HIV-DNA quantification

A minimum of 2 million peripheral blood mononuclear cells (PMBC) were thawed and resuspended in lysis buffer at a concentration of 50,000 cells/μl, and the size of proviral HIV-DNA was measured by droplet digital PCR, as previously described [89]. The total HIV-DNA estimation obtained with the QuantaSoft (BioRad) software was normalised using RPP30 housekeeping gene quantification to give a value per million PBMC. Half million cells were aliquoted for surface stain and quantification of CD4+ T-cell frequency in total PBMC. Total HIV-DNA was further adjusted for per one million CD4+ T-cells.

### Immunophenotype of peripheral NK cells

PBMC were cryopreserved in 10% dimethyl sulfoxide (DMSO, Sigma) and stored in liquid nitrogen. PBMC from 37 subjects were thawed, counted and 1 million cell per panel were rested in R10 medium for 3 hours at 37°C in 5% CO_2_ for NK cell immunophenotype. Cells were washed in PBS and stained with Live/Dead near-IR stain (Invitrogen) according to the manufacturer instructions in room temperature for 30 min. PBMC were washed again in FACS Buffer and incubated for another 30 min at 4°C in a suspension with their respective set of the fluorochrome-conjugated surface antibodies: CD14 (APC-Cy7, HCD14, Biolegend), CD19 (APC-Cy7, HIB19, Biolegend), CD3 (APC-Cy7, UCHT1, Biolegend), CD56 (BV650, 5.1H11, Biolegend), NKG2A (PE-Vio770, GL183, Miltenyi), and a cocktail of mainly inhibitory KIRs (iKIRs) on APC (KIR2DL1 143211 R&D Systems, KIR2DL2/L3/S2 GL183 Beckman Coulter, KIR3DL1 DX9 Miltenyi, KIR3DL2 539304 R&D Systems). Panel 1 also included CD16 (SB600, 3G8, Life Technologies), NKG2D (PerCP-Cy5.5, DX27, Miltenyi), NKp30 (PE-Vio615, REA823, Miltenyi), NKp46 (V450, 9E2, BD Bioscience) and DNAM-1 and (BV510, 11A8, Biolegend). Panel 2 also included NKG2C (Biotin, REA205, Miltenyi) conjugated with Streptavidin (BV570, Biolegend), CD16 (Alexa700, 3G8, Biolegend), CD57 (BV421, NK-1, BD Bioscience), CD38 (PE-Cy5, HIT2, Biolegend) and CD85j (PE, GHI/75, Biolegend). Cells for panel 2 were fixed and permeabilized with Foxp3/Transcription Factor Staining Buffer Set (eBioscience) for 45 min at 4°C and staining with the antibodies PLZF (PE-CF594, R17-809, BD Bioscience) and FcεRI-γ (FITC, Sigma-Aldrich) for 30 min at 4°C. Cells were washed and acquired on a LSR II (BD Bioscience) and data were analysed using FlowJo software v10.7.19 (TreeStar Inc.).

### Functional assays

Half to one million thawed PBMC per condition were rested in R10 medium for 5 hours for cytokine stimulation and overnight for K562 and RAJI cells assays at 37°C in 5% CO_2_. To assess cytokine stimulation, PBMC were either left unstimulated or incubated with 10 ng/mL of rhIL-12 (R&D Systems) and 100 ng/mL of rhIL18 (R&D Systems) for 18 hours at 37°C. Brefeldin A (BFA, Biolegend) and Monensin (Biolegend) were added in the last 5 hours. For K562 cells condition, PBMCs were incubated for 6 hours at 37°C with and without the target cell (5:1 effector to target ratio). For ADCC study, RAJI cells (1 million cells/mL) were coated with 2.5 μg/ml of either murine IgG or anti-CD20 (R&D Systems) for 30 min, washed and incubated with PBMC at a 10:1 effector to target ratio for 6 hours at 37°C. For both K562 and RAJI assays, cells were incubated with CD107a (APC, H4A3, BD Bioscience) and BFA/Monensin were added in the last 5 hours. At the end of incubation, cells were washed in PBS and stained with Live/Dead near-IR stain (Invitrogen) in room temperature for 30min, followed by surface staining for another 30min at 4°C with CD14 (APC-Cy7, HCD14, Biolegend), CD19 (APC-Cy7, HIB19, Biolegend), CD3 (APC-Cy7, UCHT1, Biolegend), CD56 (BV650, 5.1H11, Biolegend), CD16 (SB600, 3G8, Life Technologies), NKG2A (PE-Vio770, GL183, Miltenyi), NKG2C (PE, 134591, R&D Systems), CD57 (BV421, NK-1, BD Bioscience), a cocktail of KIRs (iKIRs) on Alexa700 (KIR2DL1 143211, KIR2DL2/L3/S2/S4 180704, KIR3DL1 177407, KIR3DL2 539304, R&D Systems). After fixed and permeabilized, the cells were stained for 45 min at 4°C with PLZF (PE-CF594, R17-809, BD Bioscience) and IFN-γ (BV421, 4S.B3, BD Bioscience) for 30 min at 4°C. Cells were washed and acquired on a BD LSR II, and data were analysed using FlowJo software v10.7.19 (TreeStar Inc.). The frequency of CD107a+ and IFN-γ+ cells are shown after subtracting the background. Boolean gating was used to analyse their frequency in CD56^dim^ NK cells expressing NKG2A, iKIRs, CD57 and PLZF.

### Data analysis and statistical methods

Graphs and statistical analysis were performed with Prism v9.0.1 (GraphPad Software) unless otherwise stated. Quantitative variables are shown using proportion, median and interquartile ranges. Student’s t-test or ANOVA followed by Tukey’s test for multiple comparisons was used to compare the groups in the genotype analyses. For FACS data, we used non-parametric tests: Kruskal-Wallis test followed by Dunn’s test for multiple comparisons when comparing groups, and Wilcoxon matched-pairs test was performed for pairwise comparisons. Spearman test was used for correlation analysis in association with a smoothing spline curve. The correlation matrix was built with the *corrplot* package in R (The R Core Team). To select relevant predicting covariates in relation to clinical parameters, a multivariate linear regression model using the penalised approach (LASSO) [90,91] was estimated. The package *glmnet* was used to obtain the optimal penalty via 10-fold cross-validation. Principally, the corresponding regression coefficient estimates can be interpreted analogously to a conventional linear model, just that they are typically substantially shrunk (and some even shrunk to zero resulting in variable selection). Cumulative ART-free data analyses are shown in a Kaplan-Meier plot, and curves were compared with Log-rank (Mantel-Cox) test for p-value and hazard ratio (HR). We used time to start ART as a result of meeting the prevailing CD4+ T-cell count WHO criteria for ART initiation (absolute and relative CD4+ T-cell count < 350 cells/mm^3^ and/or < 20%) as the primary outcome and censoring the data if ART was started in absence of immunological progression. The Cox proportional-hazard model was done using the *coxph function in R and the forest plot using ggforest*. Chi-square was used for categorical variables as appropriate and Bonferroni-Holm adjustment for pairwise comparisons. A linear mixed-effect regression model was used for the longitudinal analysis of CD4+ T-cell count and plasma HIV-RNA copies using the *lme4 package in R, with the lmer for continuous outcomes [92]. The model accounted for the random effect attributable to the age (in years) at which each donor had data available and a fixed effect estimate by HLA-Bw4 and HLA-A expression. Graphs were generated using ggplot2, and the smoothed lines are shown using LOESS*. The heatmap analysis was done using the *pheatmap* package after standardizing each parameter’s frequency data to z-score and calculating the median per group; the markers were clustered using the Euclidean method. Boolean gating analyses is shown in bar and pie charts and comparison done with the permutation using SPICE v6.0, as previously described [93], and Kruskal-Wallis test. We assumed a two-sided alpha error of 0.05.

## Supporting information

S1 Table Frequency of HLA-I and KIR genotypes. (PDF)

S2 Table HLA-A expression level models (z-score) previously published. (PDF)

S3 Table Logistic regression models in the paediatric and adult cohorts considering individual protective and disease-susceptible HLA-I alleles. (PDF)

S4 Table Individual clinical data. (PDF)

S1 Fig Individual effect of protective HLA-B*57/58:01/81:01 (A) and disease-susceptible HLA-B*18:01/45:01/58:02 (B) on time to start ART due to progression (absolute and relative CD4+ T-cell count <350 cells/mm^3^ and/or < 20%). Data was censored if ART initiated without immunological progression. p-values are based on log-rank comparisons between the presence or not of the listed HLA-I alleles.
(PDF)

S2 Fig Comparison of absolute (A) and relative (B) CD4+ T-cell count and plasma viral load (C) between PSP-PP, PSP-VNC and PSP-VC. Statistical comparison between the three groups were based on ANOVA followed by Tukey’s test for multiple comparisons.
(PDF)

S3 Fig Frequency of the individual disease-protective (A) and the disease-susceptible (B) HLA-I alleles in each group. Comparisons were based on Chi-square test and corrected with Bonferroni-Holm due to multiple-comparison tests.
(PDF)

S4 Fig Strategy analyses to gate on NK cell subsets (A) and frequency of total NK cells as a percentage of total lymphocytes (B). (PDF)

S5 Fig Frequency of surface markers shown for CD56^dim^ NK cell in the four groups. A to H. Individual surface expression of different markers. Statistical comparison between the three HIV-infected groups was based on Kruskal-Wallis test followed by Dunn’s test for multiple comparisons.
(PDF)

S6 Fig Frequency of NKG2A and NKp46 co-expression on CD56dim NK cell in the four groups. Statistical comparison between the three HIV-infected groups was based on Kruskal-Wallis test followed by Dunn’s test for multiple comparisons.
(PDF)

S7 Fig Pie and bar charts analysis performed by SPICE looking the combination of NKG2A, iKIRs, CD85j and CD57. Each pie slice corresponds to a different combination in the bar chart. The arcs represent the populations who NKG2A+ (black) and iKIRs+ (grey). Kruskal-Wallis test followed by Dunn’s test for multiple comparisons was performed to compare the groups.
(PDF)

S8 Fig Effect of HLA-B -21M/T on the frequency of NKG2A+ CD56^dim^ NK cells. (PDF)

S9 Fig Correlation of IFN-γ+ upon IL-12/IL-18 stimulation with NKG2A+ CD56dim NK cells, relative CD4+ T-cell, plasma HIV RNA and total HIV DNA loads. Spearman rank test are shown for correlations and the black line represents smoothing spline.
(PDF)
