# Supplementary material for: An HLA-I signature favouring KIR-educated Natural Killer cells mediates immune control of HIV in children and contrasts with the HLA-B-restricted CD8+ T-cell-mediated immune control in adults
Source: PLoS Pathog. 2021 Nov 18;17(11):e1010090. doi: 10.1371/journal.ppat.1010090 (PMC8639058; doi:10.1371/journal.ppat.1010090)
Supplement: S1 Table — (PDF) [file ppat.1010090.s001.pdf]

S1Table. Frequency of HLA-I and KIR genotypes

HLA-I	PP (n=73)	PSP (n=237)	*p-value*	PP (n=73)	PSP-PP (n=57)	PSP-VNC (n=112)	PSP-VC (n=68)
**pHLA**[2]	19% (14)	29% (69)	*ns*	19% (14)	23% (13)	29% (32)	35% (24)
**HLA-B*57**	3% (2)	10% (24)	*0.05*	3% (2)	9% (5)	9% (10)	13% (9)
**HLA-B*58:01**	10% (7)	11% (26)	*ns*	10% (7)	10% (6)	12% (13)	10% (7)
**HLA-B*81:01**	7% (5)	11% (26)	*ns*	7% (5)	7% (4)	9% (10)	18% (12)
**sHLA**[3]	41% (30)	35% (84)	*ns*	41% (30)	40% (23)	33% (37)	35% (24)
**HLA-B*18:01**	8% (6)	4% (10)	*ns*	8% (6)	2% (1)	5% (6)	4% (3)
**HLA-B*45:01**	16% (12)	9% (22)	*ns*	16% (12)	9% (5)	10% (11)	9% (6)
**HLA-B*58:02**	18% (13)	23% (55)	*ns*	18% (13)	30% (17)	19% (21)	25% (17)
**HLA-Bw4**	49% (36)	67% (158)	0.0087	49% (36)	68% (39)	64% (72)	69% (47)
**HLA-Bw4-80I**	38% (28)	48% (113)	*ns*	38% (28)	56% (32)	42% (47)	50% (34)
**HLA-C1**	63% (46)	57% (136)	*ns*	63% (46)	54% (31)	58% (65)	59% (40)
**HLA-C2**	81% (59)	72% (171)	*ns*	81% (59)	63% (36)	72% (81)	79% (54)

KIR	PP (n=73)	PSP (n=126)	*p-value*	PP (n=73)	PSP-PP (n=13)	PSP-VNC (n=70)	PSP-VC (n=43)
**KIR2DL1**	100% (73)	99% (125)	*ns*	100% (73)	100% (13)	99% (69)	100% (43)
**KIR2DL2**	71% (52)	66% (83)	*ns*	71% (52)	69% (9)	71% (50)	56% (24)
**KIR2DL3**	79% (58)	71% (89)	*ns*	79% (58)	54% (7)	73% (51)	72% (31)
**KIR2DL4**	100% (73)	99% (125)	*ns*	100% (73)	100% (13)	99% (69)	100% (43)
**KIR2DL5**	70% (51)	60% (76)	*ns*	70% (51)	77% (10)	63% (44)	51% (22)
**KIR2DS1**	25% (18)	17% (22)	*ns*	25% (18)	31% (4)	16% (11)	16% (7)
**KIR2DS2**	68% (50)	65% (82)	*ns*	68% (50)	69% (9)	70% (49)	56% (24)
**KIR2DS3**	23% (17)	26% (33)	*ns*	23% (17)	8% (1)	30% (21)	26% (11)
**KIR2DS4**[4]	86% (63)	87% (110)	*ns*	86% (63)	54% (7)	90% (63)	93% (40)
**KIR2DS5**	58% (42)	47% (59)	*ns*	58% (42)	69% (9)	47% (33)	40% (17)
**KIR3DL1**	100% (73)	98% (124)	*ns*	100% (73)	100% (13)	99% (69)	98% (42)
**KIR3DS1**	11% (8)	10% (12)	*ns*	11% (8)	8% (1)	9% (6)	12% (5)
**KIR3DL2**	100% (73)	99% (125)	*ns*	100% (73)	100% (13)	99% (69)	100% (43)
**KIR3DL3**	100% (73)	99% (125)	*ns*	100% (73)	100% (13)	99% (69)	100% (43)

[1]Chi-square test for trend;
[2]p-HLA: disease-protective HLA (HLA-B*57/58:01/81:01);
[3]s-HLA: disease-susceptible HLA (HLA-B*18:01/45:01/58:02);
[4]full-length gene
