# Supplementary material for: An HLA-I signature favouring KIR-educated Natural Killer cells mediates immune control of HIV in children and contrasts with the HLA-B-restricted CD8+ T-cell-mediated immune control in adults
Source: PLoS Pathog. 2021 Nov 18;17(11):e1010090. doi: 10.1371/journal.ppat.1010090 (PMC8639058; doi:10.1371/journal.ppat.1010090)
Supplement: S2 Table — (PDF) [file ppat.1010090.s002.pdf]

S2 Table. HLA-A expression level models (z-score) previously published [1].

HLA-A Lineage	**Expression estimate (z-score)**
*A*01*	0.11
*A*02*	0.11
*A*03*	-0.77
*A*11*	0.14
*A*20*	-0.37
*A*23*	0.34
*A*24*	0.94
*A*25*	-0.04
*A*26*	0.15
*A*29*	0.46
*A*30*	0.26
*A*31*	-0.46
*A*32*	-0.73
*A*33*	-0.77
*A*34*	0.37
*A*36*	0.21
*A*43*	-0.17
*A*66*	0.25
*A*68*	0.70
*A*69*	0.53
*A*74*	-1.02
*A*80*	-0.39

REFERENCE

1. Ramsuran V, Naranbhai V, Horowitz A, Qi Y, Martin MP, Yuki Y, et al. Elevated *HLA-A* expression impairs HIV control through inhibition of NKG2A expressing cells. Science. 2018;359(6371):86-90. Epub 2018/01/04. doi: 10.1126/science.aam8825. PubMed PMID: 29302013; PubMed Central PMCID: PMCPMC5933048.
