# Supplementary material for: An HLA-I signature favouring KIR-educated Natural Killer cells mediates immune control of HIV in children and contrasts with the HLA-B-restricted CD8+ T-cell-mediated immune control in adults
Source: PLoS Pathog. 2021 Nov 18;17(11):e1010090. doi: 10.1371/journal.ppat.1010090 (PMC8639058; doi:10.1371/journal.ppat.1010090)
Supplement: S3 Table — (PDF) [file ppat.1010090.s003.pdf]

S3 Table. Logistic regression models in the paediatric and adult cohorts considering individual protective and disease-susceptible HLA-I alleles.

Model 1. Outcome: PP = 1; PSP = 0

Predictor	Coefficient	Standard error	p-value	OR	95% CI of OR	
					Lower	Upper
Intercept	-1.42550	0.63540	0.025	0.24	0.06	0.79
*HLA-B*57*	-0.42900	0.82140	0.601	0.65	0.09	2.76
*HLA-B*58:01*	0.38110	0.58550	0.515	1.46	0.43	4.42
*HLA-B*81*	-1.05680	0.56550	0.062	0.35	0.10	0.98
*HLA-B*18:01*	0.64630	0.57980	0.265	1.91	0.60	5.97
HLA-B*45	0.32170	0.44200	0.467	1.38	0.57	3.24
HLA-B*58:02	0.30090	0.45430	0.508	1.35	0.55	3.29
**HLA-Bw4**	**-1.10800**	**0.41140**	**0.007**	**0.33**	**0.14**	**0.73**
HLA-B -21T	0.30290	0.60120	0.614	1.35	0.43	4.77
HLA-C C1C1	*Reference*					
C1C2	0.55810	0.47100	0.236	1.75	0.72	4.64
C2C2	0.69660	0.52680	0.186	2.01	0.73	5.89
**HLA-A (z-score)**	**0.63240**	**0.25620**	**0.014**	**1.88**	**1.16**	**3.18**

Model 2. Outcome: PP=3, PSP-PP=2, PSP-VNC=1, PSP-VC=0

Predictor	Coefficient	Standard error	p-value	OR	95% CI of OR	
					Lower	Upper
Intercept	1.43455	0.27989	<0.001	4.20	2.43	7.27
*HLA-B*57*	-0.40597	0.28743	0.159	0.67	0.38	1.17
*HLA-B*58:01*	0.05056	0.24089	0.834	1.05	0.66	1.69
HLA-B*81	**-0.61485**	**0.22609**	**0.007**	**0.54**	**0.35**	**0.84**
*HLA-B*18:01*	0.16412	0.28724	0.568	1.18	0.67	2.07
HLA-B*45	0.16588	0.21434	0.440	1.18	0.78	1.80
HLA-B*58:02	-0.07274	0.19547	0.710	0.93	0.63	1.36
**HLA-Bw4**	**-0.42616**	**0.17675**	**0.017**	**0.65**	**0.46**	**0.92**
HLA-B -21T	0.13436	0.27388	0.624	1.14	0.67	1.96
HLA-C C1C1	*Reference*					
C1C2	0.20382	0.1971	0.302	1.23	0.83	1.80
C2C2	0.21345	0.22207	0.337	1.24	0.80	1.91
**HLA-A (z-score)**	**0.34711**	**0.10176**	**0.001**	**1.41**	**1.16**	**1.73**

Model 3. Outcome: Adult-P = 2, Adult-VNC = 1, Adult-VC = 0

Predictor	Coefficient	Standard error	P-value	OR	95% CI of OR	
					Lower	Upper
Intercept	1.079563	0.084901	<0.001	2.94	2.49	3.48
HLA-B*57	**-0.300951**	**0.099949**	**0.003**	**0.74**	**0.61**	**0.90**
*HLA-B*58:01*	-0.126502	0.08845	0.153	0.88	0.74	1.05
HLA-B*81	**-0.297718**	**0.079431**	**<0.001**	**0.74**	**0.64**	**0.87**
HLA-B*18:01	**0.336877**	**0.095352**	**<0.001**	**1.40**	**1.16**	**1.69**
HLA-B*45	0.067484	0.089174	0.449	1.07	0.90	1.27
**HLA-B*58:02**	**0.310816**	**0.069619**	**<0.001**	**1.36**	**1.19**	**1.56**
**HLA-Bw4**	**-0.19336**	**0.06544**	**0.003**	**0.82**	**0.72**	**0.94**
HLA-B -21T	0.005349	0.082449	0.948	1.01	0.86	1.18
HLA-C C1C1	*Reference*					
C1C2	0.024693	0.065404	0.706	1.03	0.90	1.17
C2C2	0.015861	0.073897	0.830	1.02	0.88	1.17
**HLA-A (z-score)**	**0.083442**	**0.033594**	**0.013**	**1.09**	**1.02**	**1.16**
