# Supplementary material for: An HLA-I signature favouring KIR-educated Natural Killer cells mediates immune control of HIV in children and contrasts with the HLA-B-restricted CD8+ T-cell-mediated immune control in adults
Source: PLoS Pathog. 2021 Nov 18;17(11):e1010090. doi: 10.1371/journal.ppat.1010090 (PMC8639058; doi:10.1371/journal.ppat.1010090)
Supplement: S4 Table — (PDF) [file ppat.1010090.s004.pdf]

**S4 Table.** Individual clinical data.

| PID | Sex | Age (years) | HLA class I | | | | | | | Plasma HIV-RNA (copies /mL) | Absolute CD4+ T-cell (count/mL) | Relative CD4+ T-cell (%) | CD4:CD8 ratio | CMV IgG (AU/mL) |
|---|---|---|---|---|---|---|---|---|---|---|---|---|---|---|
| | | | HLA-A | | HLA-A z-score | HLA-B | | HLA-C | | | | | | |
| PSP-PP-1 | Female | 10.47 | 30:01 | 66:01 | 0.44321 | 39:10 | 42:01 | 12:03 | 17:01 | 19,000 | 297 | 12 | 0.18 | >250 |
| PSP-PP-2 | Male | 9.73 | | | | *Not available* | | | | 210,000 | 55 | 3 | 0.12 | 126.1 |
| PSP-PP-3 | Male | 9.25 | | | | *Not available* | | | | 820,000 | 218 | 3 | | 244.7 |
| PSP-PP-4 | Male | 8.22 | 02:05 | 66:01 | -0.03258 | 14:01 | 58:02 | 06:02 | 08:04 | 44,000 | 164 | 7 | 0.16 | 65.9 |
| PSP-PP-5 | Female | 9.55 | 24:02 | 68:02 | 1.21040 | 07:02 | 15:10 | 07:02 | 08:04 | 470,000 | 86 | 7 | 0.10 | 137.7 |
| PSP-PP-6 | Male | 7.46 | 02:05 | 74:01 | -1.28629 | 15:03 | 58:01 | | 07:01 | 54,000 | 251 | 9 | 0.28 | >250 |
| PSP-PP-7 | Female | 10.24 | 02:02 | 30:02 | 0.07651 | 08:01 | 57:03 | 07:01 | 07:01 | 3,700,000 | 112 | 2 | | 126.5 |
| PSP-PP-8 | Female | 8.65 | 02:01 | 30:02 | 0.07651 | 45:01 | 53:01 | 04:01 | 16:01 | 740,000 | 242 | 9 | 0.12 | >250 |
| PSP-PP-9 | Female | 9.91 | 30:01 | 33:03 | -0.62485 | 07:02 | 42:01 | 07:02 | 17:01 | 590,000 | 41 | 3 | 0.08 | 231.1 |
| PSP-PP-10 | Male | 8.47 | 02:01 | 03:01 | -1.04272 | 58:01 | 58:02 | 03:02 | 06:02 | 57,000 | 321 | 8 | 0.36 | 109.2 |
| PSP-VNC-1 | Male | 10.12 | 30:02 | 68:02 | 0.59850 | 15:10 | 42:01 | 03:04 | 17:01 | 57,000 | 933 | 45 | 1.69 | 198.2 |
| PSP-VNC-2 | Female | 11.88 | 02:05 | 29:00 | 0.15346 | 42:01 | 44:03 | 02:02 | 17:01 | 110,000 | 629 | 38 | 1.24 | >250 |
| PSP-VNC-3 | Male | 11.88 | 02:01 | 26:01 | -0.05353 | 08:01 | 41:01 | 07:01 | 17:01 | 23,000 | 721 | 28 | 0.81 | 173 |
| PSP-VNC-4 | Male | 12.72 | 03:01 | 23:01 | -0.59330 | 13:03 | 44:03 | 03:03 | 06:02 | 58,000 | 1345 | 25 | 0.53 | 68 |
| PSP-VNC-5 | Female | 13.69 | 02:01 | 34:02 | 0.13538 | 44:03 | 45:01 | 04:01 | 16:01 | 24,000 | 1051 | 37 | 0.90 | 12.2 |
| PSP-VNC-6 | Female | 10.19 | 29:02 | 30:02 | 0.62925 | 14:02 | 57:03 | 08:02 | 18:02 | 140,000 | 954 | 34 | 0.89 | 66.7 |
| PSP-VNC-7 | Female | 10.50 | 02:14 | 03:01 | -1.04272 | 44:03 | 58:02 | 04:01 | 06:02 | 18,000 | 715 | 25 | 0.44 | >250 |
| PSP-VNC-8 | Male | 11.34 | 29:02 | 74:01 | -0.73354 | 15:03 | 44:03 | 02:10 | 07:01 | 27,000 | 375 | 18 | 0.36 | - |
| PSP-VC-1 | Female | 12.75 | 03:01 | 68:02 | -0.52073 | 14:02 | 58:02 | 06:02 | 08:02 | 50 | 700 | 35 | 1.13 | 186.4 |
| PSP-VC-2 | Female | 11.77 | 03:01 | 30:02 | -0.56692 | 14:01 | 15:10 | 03:04 | 08:02 | 25 | 1258 | 37 | 1.00 | 72.5 |
| PSP-VC-3 | Female | 13.58 | 03:01 | 33:03 | -1.74409 | 35:01 | 53:01 | 04:01 | 16:01 | 26 | 1316 | 39 | 2.01 | 93.3 |
| PSP-VC-4 | Female | 12.00 | 02:01 | 74:01 | -1.28629 | 27:03 | 42:01 | 02:02 | 07:01 | 35 | 768 | 32 | 0.88 | 242.3 |
| PSP-VC-5 | Female | 11.54 | 30:09 | 68:01 | 0.59850 | 58:02 | 81:01 | 04:01 | 06:02 | 1,800 | 902 | 39 | 0.86 | 105.7 |
| PSP-VC-6 | Female | 11.90 | 29:02 | 66:01 | 0.52016 | 39:10 | 44:03 | 07:01 | 12:03 | 430 | 1007 | 38 | 1.18 | 30.6 |
| PSP-VC-7 | Male | 12.86 | 02:05 | 68:02 | 0.12270 | 14:01 | 15:10 | 03:04 | 08:04 | 1,700 | 982 | 33 | 1.63 | >250 |
| PSP-VC-8 | Male | 13.50 | 02:05 | 30:01 | 0.07651 | 58:01 | 81:03 | 07:01 | 08:04 | 20 | 1007 | 34 | 1.37 | >250 |
| PSP-VC-9 | Female | 12.61 | | | | *Not available* | | | | 1,000 | 819 | 38 | 1.67 | - |
| PSP-VC-10 | Male | 12.68 | | | | *Not available* | | | | 1,200 | 665 | 34 | 0.88 | 32.8 |
| HEU-1 | Male | 12.33 | | | | *Not available* | | | | - | - | - | - | 47.2 |
| HEU-2 | Female | 11.72 | | | | *Not available* | | | | - | - | - | - | 167.7 |
| HEU-3 | Male | 12.11 | | | | *Not available* | | | | - | - | - | - | >250 |
| HEU-4 | Female | 13.12 | | | | *Not available* | | | | - | - | - | - | 226.6 |
| HEU-5 | Male | 10.27 | | | | *Not available* | | | | - | - | - | - | 95.5 |
| HEU-6 | Female | 11.55 | | | | *Not available* | | | | - | - | - | - | 72.2 |
| HEU-7 | Female | 11.14 | | | | *Not available* | | | | - | - | - | - | >250 |
| HEU-8 | Female | 10.61 | | | | *Not available* | | | | - | - | - | - | 109.6 |
| HEU-9 | Male | 13.23 | | | | *Not available* | | | | - | - | - | - | 138.8 |
