# Supplementary material for: An HLA-I signature favouring KIR-educated Natural Killer cells mediates immune control of HIV in children and contrasts with the HLA-B-restricted CD8+ T-cell-mediated immune control in adults
Source: PLoS Pathog. 2021 Nov 18;17(11):e1010090. doi: 10.1371/journal.ppat.1010090 (PMC8639058; doi:10.1371/journal.ppat.1010090)
Supplement: S1 Fig — Data was censored if ART initiated without immunological progression. p-values are based on log-rank comparisons between the presence or not of the listed HLA-I alleles. (PDF) [file ppat.1010090.s005.pdf]

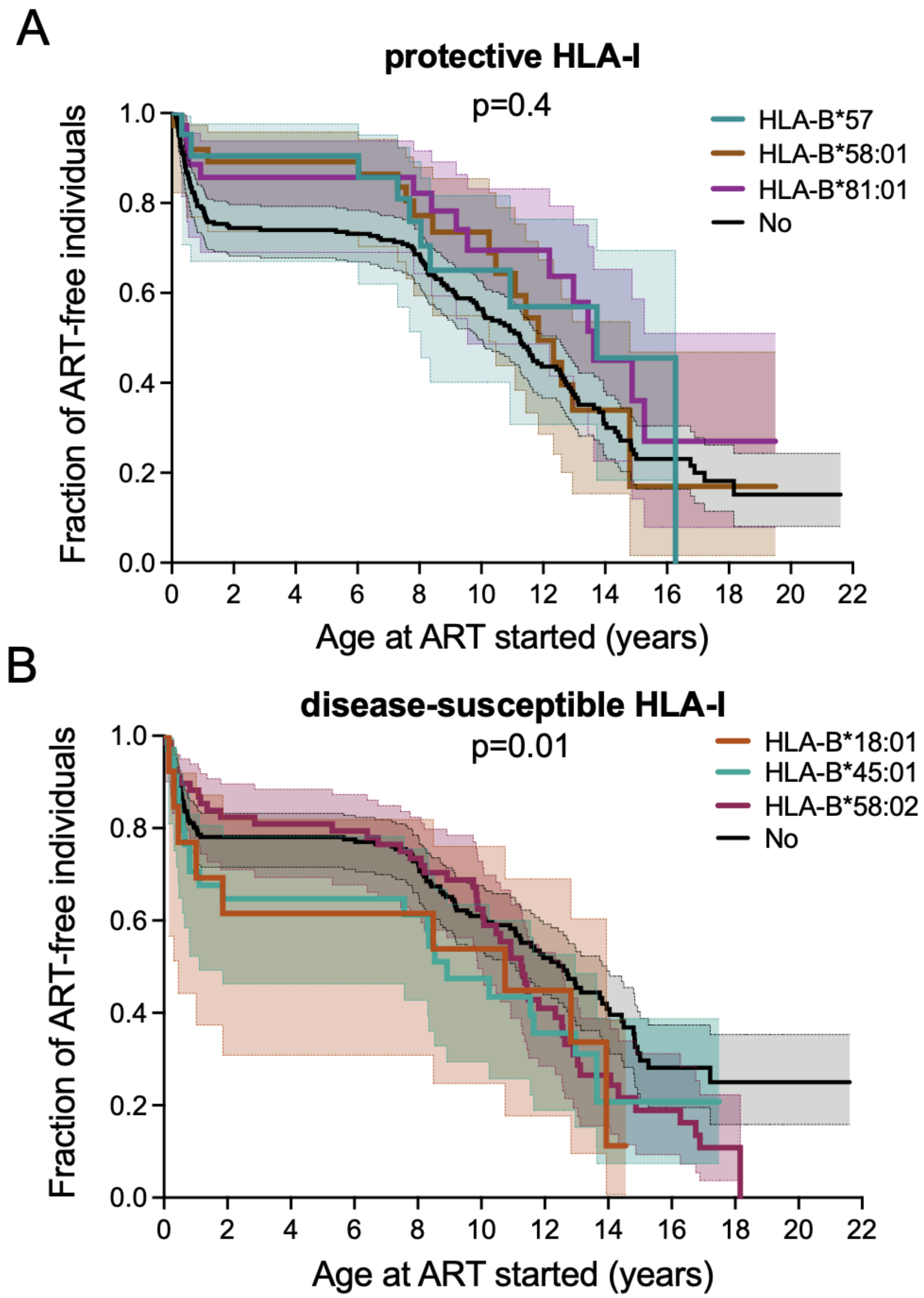

S1 Fig. Individual effect of protective HLA-B*57/58:01/81:01 (A) and disease-susceptible HLA-B*18:01/45:01/58:02 (B) on time to start ART due to progression (absolute and relative CD4+ T-cell count <350 cells/mm^3 and/or < 20%). Data was censored if ART initiated without immunological progression. p-values are based on log-rank comparisons between the presence or not of the listed HLA-I alleles.
