# Supplementary material for: An HLA-I signature favouring KIR-educated Natural Killer cells mediates immune control of HIV in children and contrasts with the HLA-B-restricted CD8+ T-cell-mediated immune control in adults
Source: PLoS Pathog. 2021 Nov 18;17(11):e1010090. doi: 10.1371/journal.ppat.1010090 (PMC8639058; doi:10.1371/journal.ppat.1010090)
Supplement: S2 Fig — Statistical comparison between the three groups were based on ANOVA followed by Tukey’s test for multiple comparisons. (PDF) [file ppat.1010090.s006.pdf]

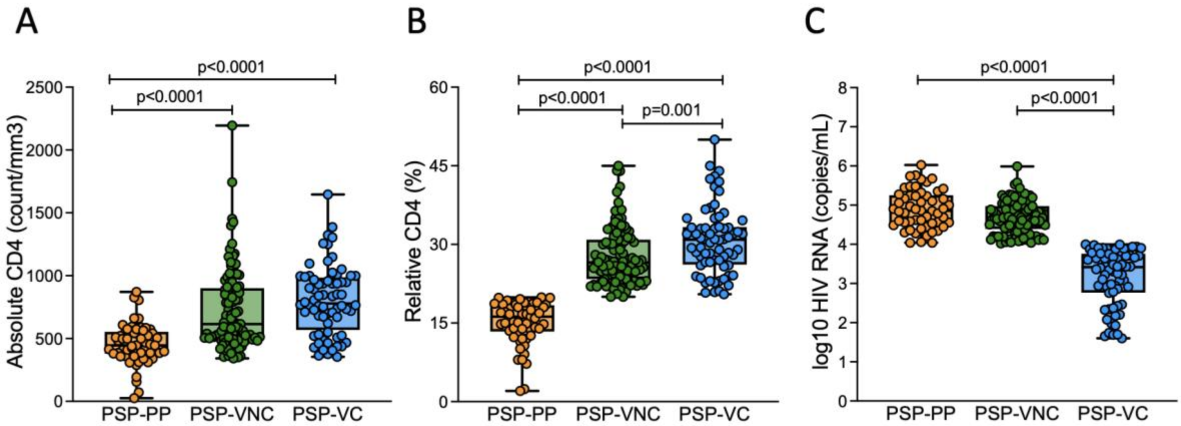

S2 Fig. Comparison of absolute (A) and relative (B) CD4+ T-cell count and plasma viral load (C) between PSP-PP, PSP-VNC and PSP-VC. Statistical comparison between the three groups were based on ANOVA followed by Tukey's test for multiple comparisons.
