# Supplementary material for: An HLA-I signature favouring KIR-educated Natural Killer cells mediates immune control of HIV in children and contrasts with the HLA-B-restricted CD8+ T-cell-mediated immune control in adults
Source: PLoS Pathog. 2021 Nov 18;17(11):e1010090. doi: 10.1371/journal.ppat.1010090 (PMC8639058; doi:10.1371/journal.ppat.1010090)
Supplement: S3 Fig — Comparisons were based on Chi-square test and corrected with Bonferroni-Holm due to multiple-comparison tests. (PDF) [file ppat.1010090.s007.pdf]

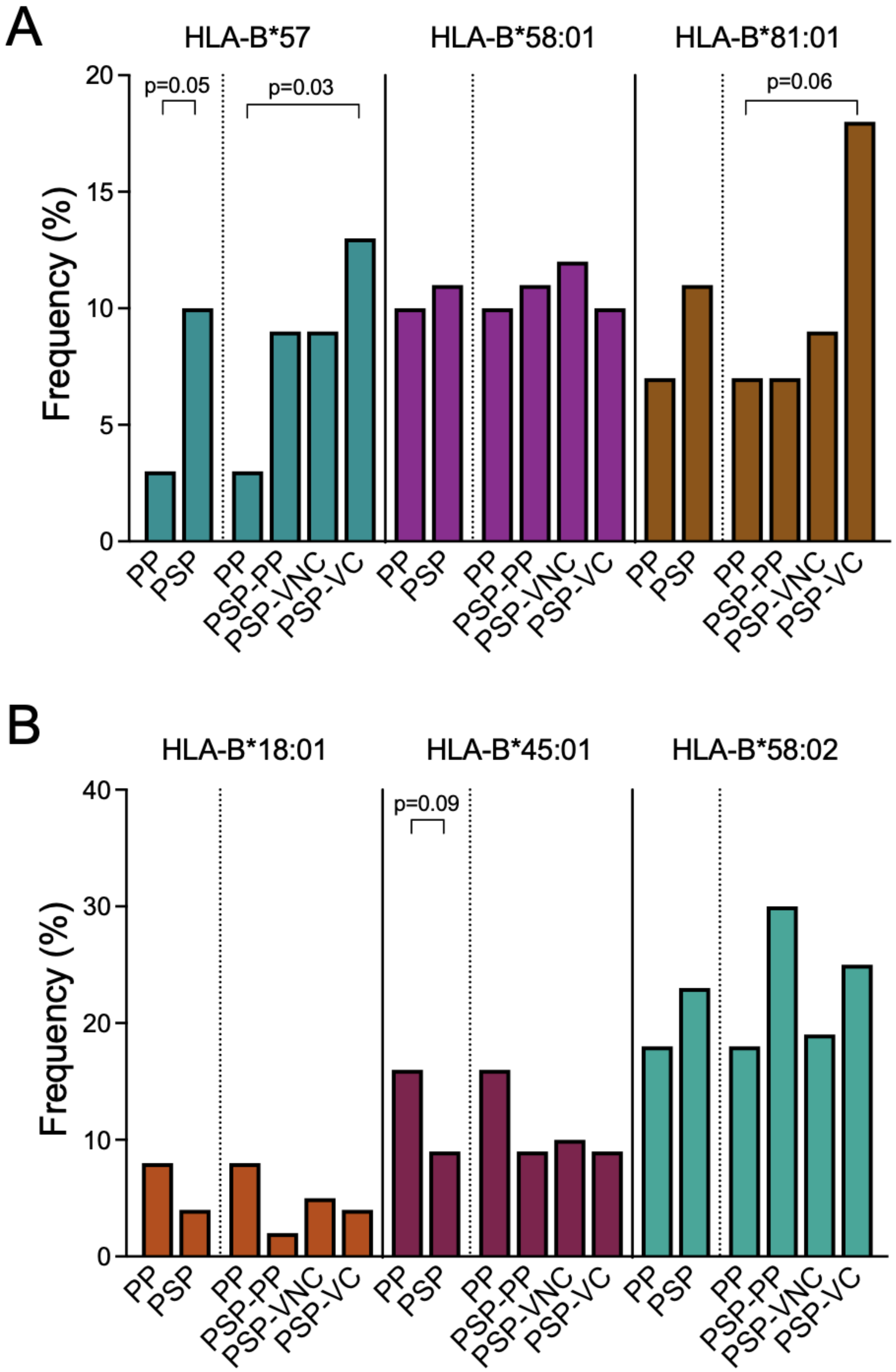


**S3 Fig.** Frequency of the individual disease-protective (A) and the disease-susceptible (B) HLA-I alleles in each group. Comparisons were based on Chi-square test and corrected with Bonferroni-Holm due to multiple-comparison tests.
