# Supplementary material for: An HLA-I signature favouring KIR-educated Natural Killer cells mediates immune control of HIV in children and contrasts with the HLA-B-restricted CD8+ T-cell-mediated immune control in adults
Source: PLoS Pathog. 2021 Nov 18;17(11):e1010090. doi: 10.1371/journal.ppat.1010090 (PMC8639058; doi:10.1371/journal.ppat.1010090)
Supplement: S4 Fig — (PDF) [file ppat.1010090.s008.pdf]

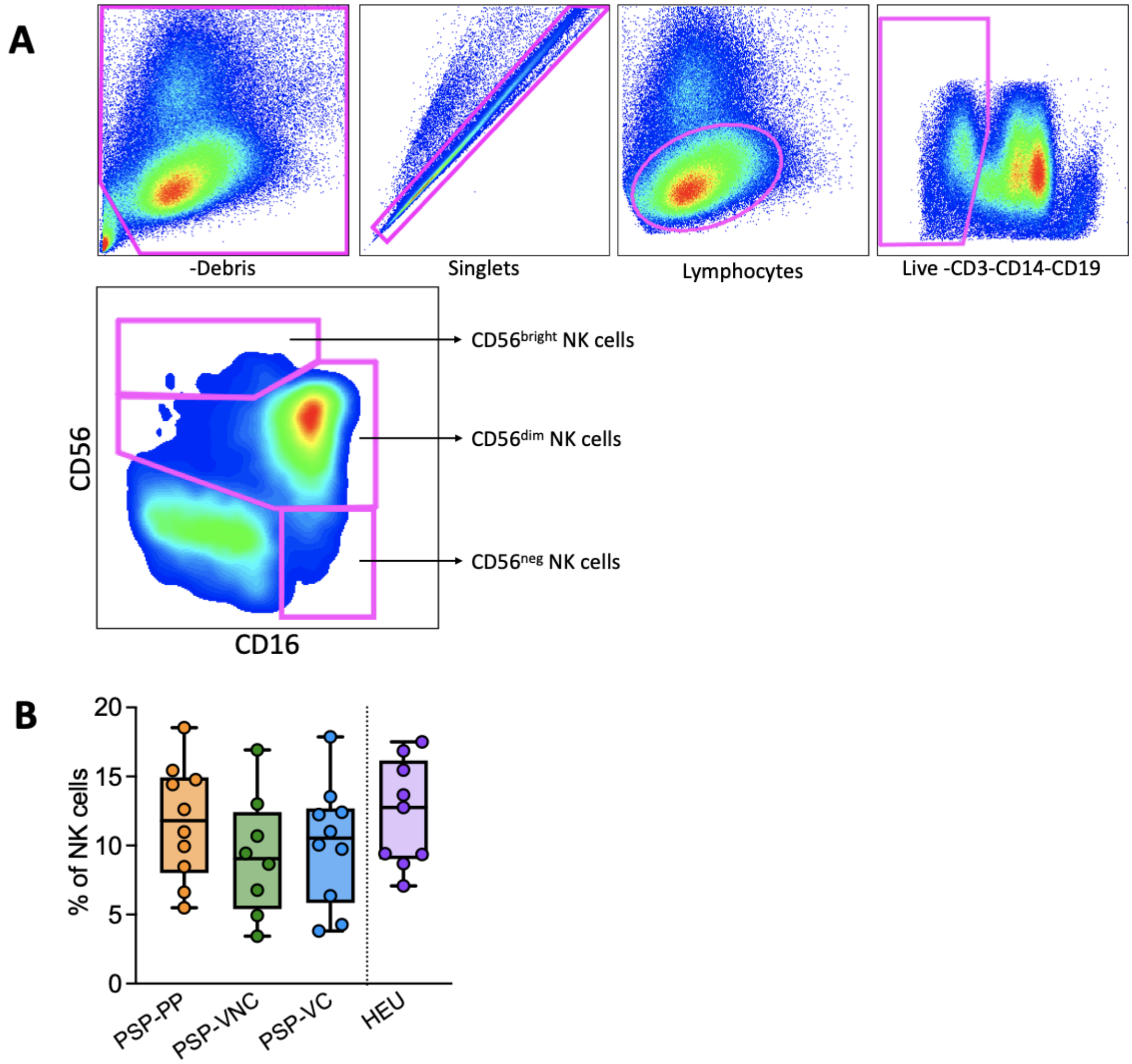

S4 Fig. Strategy analyses to gate on NK cell subsets (A) and frequency of total NK cells as a percentage of total lymphocytes (B).
