# Supplementary material for: An HLA-I signature favouring KIR-educated Natural Killer cells mediates immune control of HIV in children and contrasts with the HLA-B-restricted CD8+ T-cell-mediated immune control in adults
Source: PLoS Pathog. 2021 Nov 18;17(11):e1010090. doi: 10.1371/journal.ppat.1010090 (PMC8639058; doi:10.1371/journal.ppat.1010090)
Supplement: S6 Fig — Statistical comparison between the three HIV-infected groups was based on Kruskal-Wallis test followed by Dunn’s test for multiple comparisons. (PDF) [file ppat.1010090.s010.pdf]

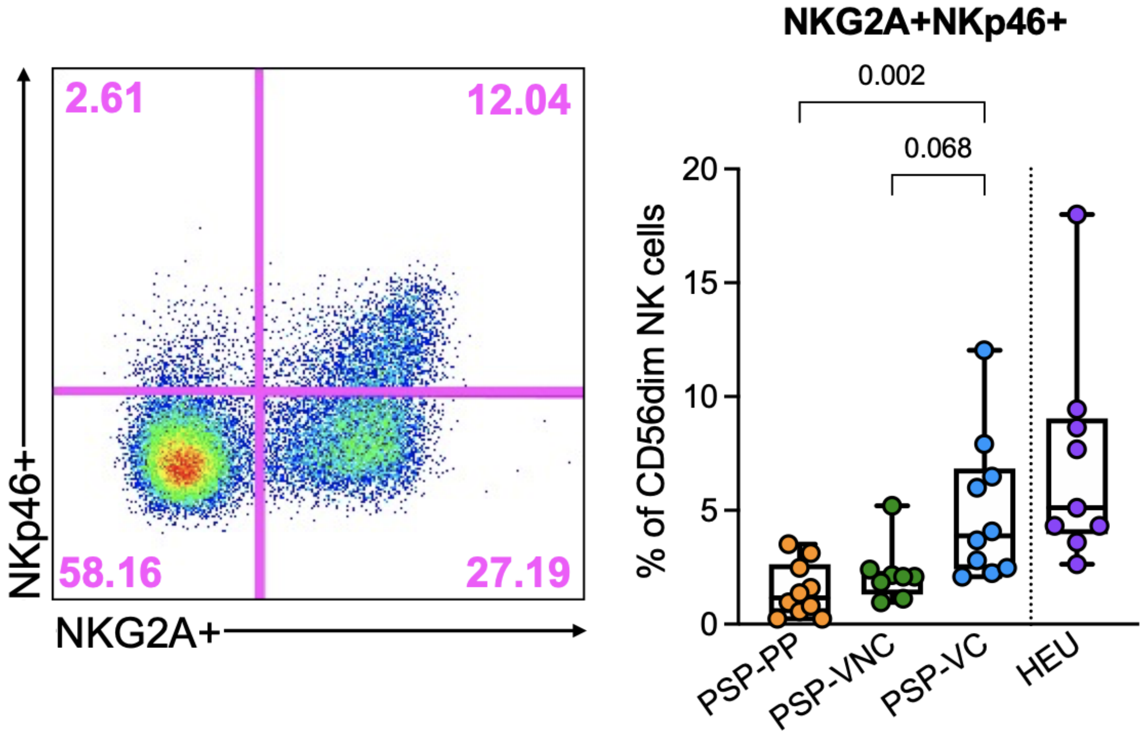

S6 Fig. Frequency of NKG2A and NKp46 co-expression on CD56dim NK cell in the four groups. Statistical comparison between the three HIV-infected groups was based on Kruskal-Wallis test followed by Dunn's test for multiple comparisons.
