# Supplementary material for: An HLA-I signature favouring KIR-educated Natural Killer cells mediates immune control of HIV in children and contrasts with the HLA-B-restricted CD8+ T-cell-mediated immune control in adults
Source: PLoS Pathog. 2021 Nov 18;17(11):e1010090. doi: 10.1371/journal.ppat.1010090 (PMC8639058; doi:10.1371/journal.ppat.1010090)
Supplement: S9 Fig — Spearman rank test are shown for correlations and the black line represents smoothing spline. (PDF) [file ppat.1010090.s013.pdf]

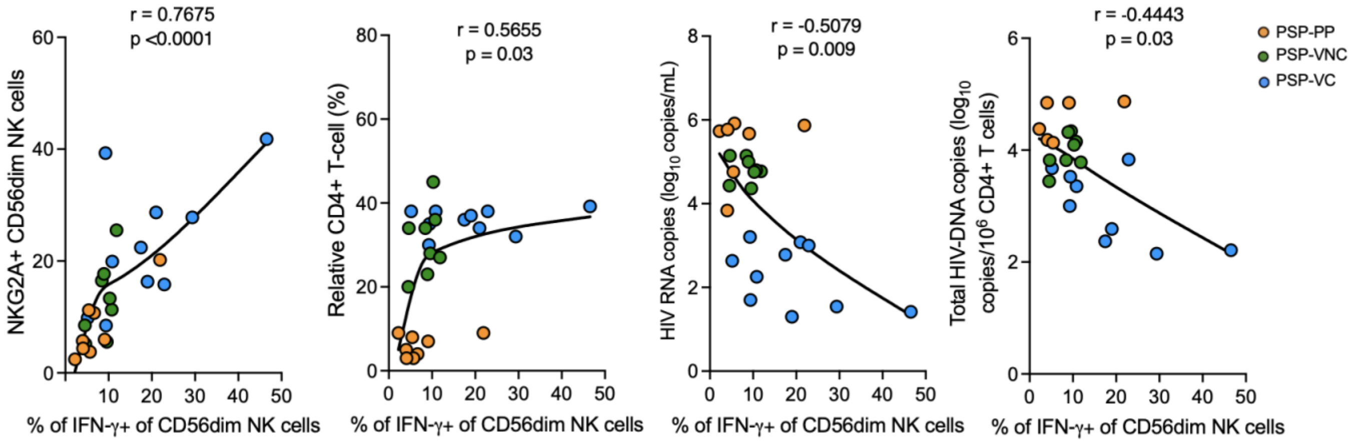

S9 Fig. Correlation of IFN-γ+ upon IL-12/IL-18 stimulation with NKG2A+ CD56dim NK cells, relative CD4+ T-cell, plasma HIV RNA and total HIV DNA loads. Spearman rank test are shown for correlations and the black line represents smoothing spline.
