# Supplementary material for: An HLA-I signature favouring KIR-educated Natural Killer cells mediates immune control of HIV in children and contrasts with the HLA-B-restricted CD8+ T-cell-mediated immune control in adults
Source: PLoS Pathog. 2021 Nov 18;17(11):e1010090. doi: 10.1371/journal.ppat.1010090 (PMC8639058; doi:10.1371/journal.ppat.1010090)
Supplement: S7 Fig — Each pie slice corresponds to a different combination in the bar chart. The arcs represent the populations who NKG2A+ (black) and iKIRs+ (grey). Kruskal-Wallis test followed by Dunn’s test for multiple comparisons was performed to compare the groups. (PDF) [file ppat.1010090.s011.pdf]

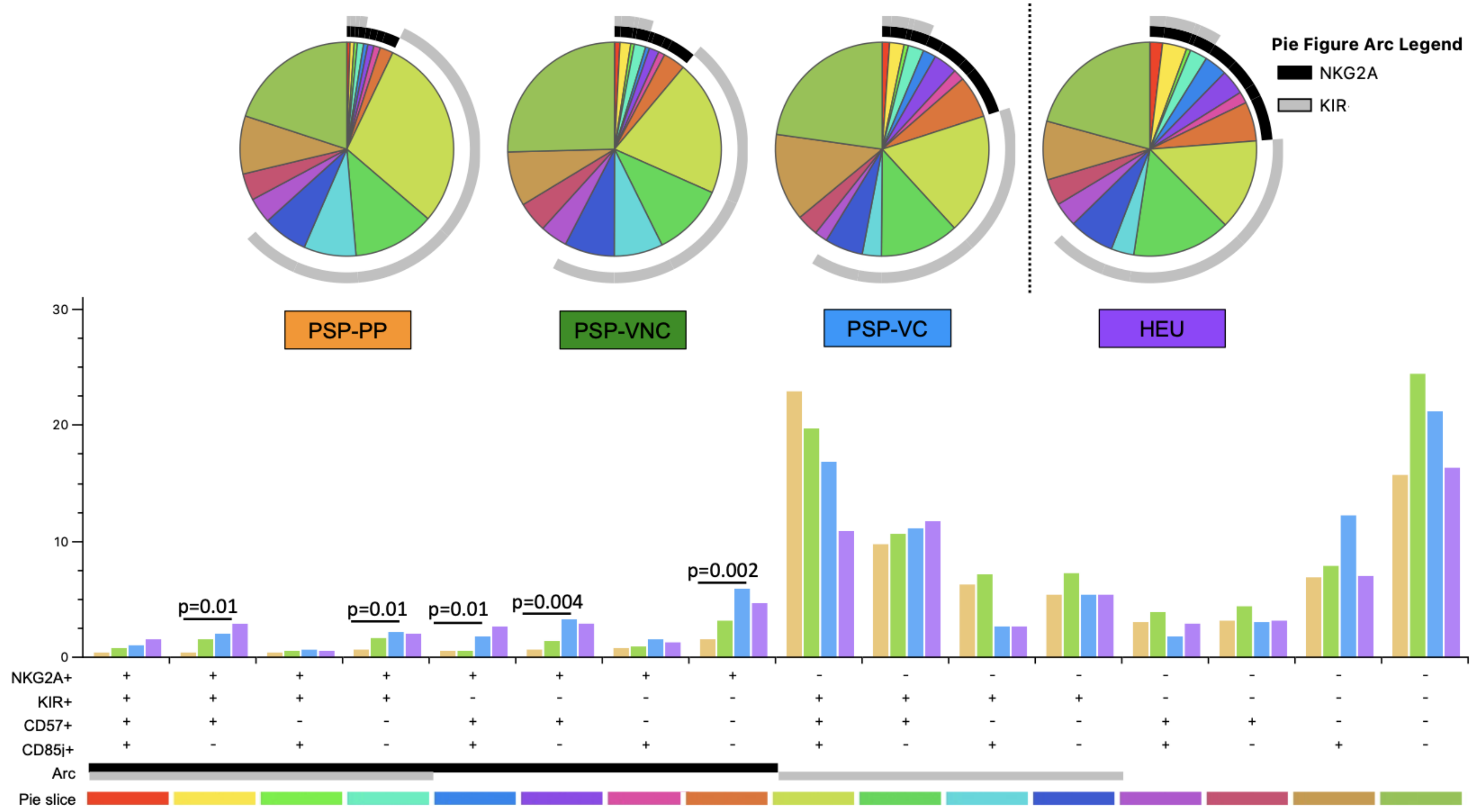

S7 Fig. Pie and bar charts analysis performed by SPICE looking the combination of NKG2A, iKIRs, CD85j and CD57. Each pie slice corresponds to a different combination in the bar chart. The arcs represent the populations who NKG2A+ (black) and iKIRs+ (grey). Kruskal-Wallis test followed by Dunn's test for multiple comparisons was performed to compare the groups.
